# Multi-Omics Reveals Light-Quality-Dependent Phytohormone and Transcription Factor Networks Regulating Flavonoid Biosynthesis in *Ludisia discolor*

**DOI:** 10.3390/genes17040445

**Published:** 2026-04-13

**Authors:** Mingyue Qiu, Yuman Shi, Tiankai Shen, Kunxiu Cai, Luan Li, Xiaoyue Qiu, Tao Zheng, Ying Chen

**Affiliations:** 1 College of Landscape Architecture and Art, Fujian Agriculture and Forestry University, Fuzhou 350100, China; 2 Fujian Institute of Tropical Crops, Zhangzhou 363001, China; 3 College of Life Sciences, Guangxi Normal University, Guilin 541006, China

**Keywords:** *Ludisia discolor*, metabolome, transcriptome, phytohormones, flavonoids

## Abstract

**Background/Objectives**: *Ludisia discolor*, an endangered medicinal orchid, is a vital source of bioactive flavonoids which requires in vitro tissue culture for propagation and metabolite production. While light quality influences metabolic processes, the mechanisms connecting light conditions, phytohormone signaling, and flavonoid biosynthesis remain unclear. This study investigates how specific light qualities trigger secondary metabolism to improve tissue culture and conservation strategies. **Methods**: *L. discolor* was cultivated under strictly regulated LED environments (blue, red, yellow, and green). An integrated multi-omics approach, combining transcriptomic sequencing and targeted metabolomic profiling, was employed to analyze leaves, correlating plant hormone changes with flavonoid metabolite levels. **Results**: LED light qualities significantly altered flavonoid and phytohormone profiles, yielding 80 unique flavonoids. Blue and red light effectively promoted flavonoid accumulation, whereas yellow light suppressed it. Transcriptomics, validated by qRT-PCR, revealed distinct expression patterns in key structural genes (e.g., *4CL*, *PAL*, *CYP73A*, *FLS*, *CCoAOMT*, *C12RT1*). Ten transcription factors (including MYB93, bZIP36, bHLH4, and bZIP44) with hormone-responsive cis-elements were co-expressed with 16 structural genes. Notably, blue light induced reactive oxygen species (ROS) signaling, activating phytohormone production (IAA, GA, ABA). These hormones subsequently stimulated transcription factors, increasing the biosynthesis of compounds like neohesperidin and hesperetin. **Conclusions**: We propose a novel regulatory model where light-induced ROS and phytohormone cascades activate specific transcription factors, enhancing structural gene expression in the flavonoid pathway. These findings elucidate the molecular mechanisms of light-driven secondary metabolism, providing valuable insights for the sustainable agriculture and ex situ conservation of endangered medicinal orchids.

## 1. Introduction

*Ludisia discolor* is a perennial herb in the genus *L. discolor* (Ludisia A. Rich.) of the family Orchidaceae [1]. Because of its root creeping and obvious stem section, which resemble a silkworm lying on stone, it is also known as “stone silkworm” or “stone lotus root” [2]. It is classified as a national second-grade protected plant [3] in China, where it is mainly distributed in Fujian, Guangdong, Hainan, and other southeastern regions [4]. The leaves of *L. discolor* are obovate in shape, have a velvet-like texture, a black-green coloration with clear veins on the top surface, and a light red/dark purple coloration on the bottom surface. This plant has a beautiful form and long-lasting vitality, making it a good ornamental plant [5]. In addition, *L. discolor* has a long medicinal history, with the effects of nourishing Yin and moistening the lungs, clearing heat and cooling the blood, invigorating the spleen, and tranquilizing the mind. In recent years, *L. discolor* has received widespread attention for its potential ornamental and medicinal values. However, intense market demand and illegal harvesting have severely depleted its natural populations, exacerbating its endangered status. To mitigate these ecological constraints and meet escalating commercial demands, in vitro tissue culture has emerged as an indispensable biotechnological strategy. This technique not only aids in germplasm conservation but also significantly increases the propagation efficiency, offering a highly controlled environment for the rapid, large-scale production of high-quality plant biomass.

The pharmacological benefits and long medicinal history of *L. discolor* mentioned above are largely attributed to its secondary metabolites, particularly flavonoids. Flavonoids represent a crucial group of polyphenolic secondary metabolites in plants, fundamentally characterized by a C6-C3-C6 phenylchromone core skeleton. These phytochemicals are ubiquitously distributed across various botanical structures, including seeds, leaves, fruits, and floral organs [6]. They are biosynthesized via the phenylpropane biosynthesis and flavonoid biosynthesis pathways. The downstream part of the flavonoid biosynthesis pathway is branched, leading to the production of various subclasses such as flavanols and anthocyanins [7]. Due to their unique chemical structures, flavonoid metabolites have pharmacological activities, such as anti-inflammatory, antibacterial, anti-tumor, hypolipidemic, cholesterol-lowering, and hepatoprotective effects [8,9]. Flavonoids act as plant antitoxins or antioxidants, owing to their ability to scavenge reactive oxygen species (ROS) [10], and protect plants from damage caused by biotic and abiotic stresses including UV irradiation, cold stress, pathogen infection, and insect feeding [11,12]. In plants, flavonoids also act as signaling molecules, are involved in plant secondary metabolism, and have multiple functions in plant stress tolerance [13,14]. For example, the overexpression of *F3H*, a key gene involved in the metabolic biosynthesis of flavonoids, leads to the overaccumulation of *Oryza sativa* L. kamaferol and quercetin, which can alleviate the accumulation of ROS under drought and ultraviolet radiation stress [15]. Moreover, key genes involved in the biosynthesis of flavonoid metabolites, such as *PAL*, *FLS*, and *F3′H*, increase the flavonoid content in plants such as *Catharanthus roseus* [16] and *Dendrobium catenatum* [17], increasing their antioxidant properties and thereby significantly enhancing the resistance of these plants to salt, light, and chilling injury. Inducing abiotic stress to promote flavonoid accumulation in plants represents a promising approach for enhancing their therapeutic value.

Phytohormones (PHs), also called endogenous plant hormones, are a group of organic compounds produced by plant cells upon receiving specific environmental signals. They trigger physiological effects in very minute concentrations and play regulatory roles in many plant life processes [18]. The primary classes of phytohormones encompass abscisic acid (ABA), auxins (AUX), brassinosteroids (BRs), cytokinins (CK), ethylene, gibberellins (GAs), jasmonic acid (JA), salicylic acid (SA), and strigolactones (SLs). By modulating internal nucleic acids, proteins, and enzymes, these signaling molecules orchestrate vital physiological events—such as plant development, metabolism, and senescence—while simultaneously directing the biosynthesis of secondary metabolites like alkaloids, flavonoids, and terpenoids [19,20]. Gibberellins, synthesized via the MEP (mevalonate) pathway, inhibit the expression of the key enzyme of the MEP pathway *DXR* (Abstract1-Deoxy-d-xylulose-5-phosphate reductoisomerase) in *Artemisia annua*, thereby inhibiting their production and resulting in lower artemisinin production [21]. Following JA elicitation, Hibiscus sabdariffa cultures exhibited an increased accumulation of bioactive compounds, alongside enhanced antioxidant capacity [22]. *Camellia vietnamensis* [23] and *pear calli* [24] showed high expression of key genes involved in flavonoid synthesis after Me JA treatment. The regulatory network of plant phytohormones, which acts on regulatory genes, further controlling structural genes, and ultimately causing the accumulation of plant secondary metabolites, is complex. However, the regulatory interplay between phytohormones and the secondary metabolites of flavonoids in the leaves of *L. discolor* is still unclear. Consequently, elucidating the regulatory mechanisms underlying foliar flavonoid biosynthesis, alongside characterizing the interplay between phytohormones and secondary metabolites, is of paramount importance for *L. discolor*.

In tissue culture systems, these internal phytohormone responses and subsequent metabolite accumulation are highly dependent on external environmental cues, with light being the most fundamental energy source driving plant survival and growth. In tissue culture systems, where plants are isolated from natural ecological cues, artificial light serves as a highly critical regulatory factor, governing photosynthesis, photomorphogenesis, and the accumulation of secondary metabolites. LEDs (light-emitting diodes) are a novel type of energy-saving light source, the light quality from which can be finely modulated in response to precise research and cultivation needs. Because artificial light quality directly dictates the success of tissue culture, utilizing LEDs is essential for optimizing in vitro environments and maximizing both plant yields and metabolite concentrations [25]. Within the visible spectrum, blue and red wavelengths serve as the primary energy drivers for photosynthetic CO2 assimilation. Consequently, these specific light qualities profoundly modulate overall plant development, cellular differentiation, and secondary metabolism [26]. In Anoectochilus roxburghii, the application of blue and yellow wavelengths optimally promotes overall plant development alongside the biosynthesis of total flavonoids and polyphenols [27]. In *Arabidopsis thaliana*, red/far-red light and blue light irradiation increase anthocyanin content. Zlatić [28] concluded that blue light could promote plant anthocyanin synthesis. Therefore, using LED light as a primary plant light source to explore the regulatory relationship between light quality and the secondary metabolites of *L. discolor* leaves is conducive to uncovering the impacts of light environmental factors on plant secondary metabolism, and is of paramount importance for the optimized tissue culture and scientific production of *L. discolor*.

As a national second-grade protected plant, *L. discolor* is highly prized for its profound medicinal properties—particularly its rich bioactive flavonoid content—and its significant economic value. However, intense market demand and habitat depletion have severely threatened its wild populations, making in vitro tissue culture an urgent necessity for its sustainable conservation and commercial application. The complex molecular mechanisms linking specific light conditions, endogenous phytohormone signaling, and downstream flavonoid biosynthesis during cultivation remain poorly understood. Therefore, the primary objective of this study is to elucidate how different light qualities regulate these interconnected networks to enhance targeted metabolite accumulation. By identifying the key regulatory nodes governing light-driven flavonoid biosynthesis, this study aims to provide a robust theoretical foundation for optimizing indoor cultivation strategies and exogenous hormone applications, ultimately facilitating the ex situ conservation and sustainable commercial production of this endangered medicinal orchid.

## 2. Materials and Methods

### 2.1. Plant Material

*L. discolor* is an endangered perennial herb natively distributed in southeastern China. Botanically, it exhibits a creeping habit with distinctive obovate, velvet-like leaves that are blackish-green with prominent veins on the adaxial (upper) surface and reddish-purple on the abaxial (lower) surface [5]. The plant material consisted of *L. discolor* ‘Min Hot Round Shuai’ seedlings, provided by the experimental base of the Fujian Tropical Crops Research Institute (Zhangzhou, Fujian, China; 117.31° E, 24.38° N). ‘Min Hot Round Shuai’ is a new variety of *L. discolor* officially registered in Fujian Province. The experiments were conducted in the tissue culture laboratory of the institute. Geographically, the institute is situated in a region with a south subtropical maritime monsoon climate and an average annual temperature of 21.1 °C.

Uncontaminated, two-month-old *L. discolor* tissue culture plantlets were selected for LED light quality treatments. The experiment comprised five light treatments: blue light (B; peak wavelength = 469 nm), red light (R; peak wavelength = 630 nm), yellow light (Y; peak wavelength = 592 nm), green light (G; peak wavelength = 519 nm), and white light (W; cool white, blue chip peak at 452 nm). White light served as the control (CK). The photosynthetic photon flux density (PPFD) was uniformly maintained at 30 μmol·m^−2^·s^−1^ (equivalent to an illuminance of 2000 lx) across all groups. LED beads were evenly distributed to ensure uniform illumination. The distance between the light sources and plants was approximately 40 cm, and different treatments were isolated using 100% opaque blackout cloth to prevent light interference. The lighting equipment was provided by Xinkangneng Optoelectronics Technology Co., Ltd. (Zhangzhou High-tech Zone, Zhangzhou, China). A total of 125 *L. discolor* plants were used, evenly distributed across the five treatments (25 plants per treatment). Cultivation occurred under highly controlled environmental conditions, featuring a photoperiod of 8 h/d and a constant temperature of 24 ± 1 °C. After 90 days of continuous cultivation, fresh leaves were collected from robust plants exhibiting uniform growth. To ensure strict sample consistency, five leaves were harvested from the same physiological position on each plant, immediately frozen in liquid nitrogen for 15 min, and subsequently stored at −80 °C pending integrated metabolomic and transcriptomic analyses.

### 2.2. Metabolomics Analysis

#### 2.2.1. Metabolite Extraction

Approximately 50 mg of *L. discolor* leaf tissue was homogenized in 1000 μL of extraction buffer (methanol: acetonitrile: water = 2:2:1, *v*/*v*/*v*) spiked with a 20 mg/L internal standard. After 30 s of vortex agitation, steel beads were introduced, and the mixture was mechanically disrupted using a tissue grinder at 45 Hz for 10 min. This was followed by 10-min sonication in an ice-water bath and subsequent incubation at −20 °C for 1 h. Following centrifugation (12,000 rpm, 4 °C, 15 min), a 500 μL aliquot of the resulting supernatant was transferred to a fresh microcentrifuge tube and evaporated to dryness using a vacuum concentrator. For metabolite reconstitution, the dried pellet was resuspended in 160 μL of a 1:1 (*v*/*v*) acetonitrile/water solution. The samples were vortexed for 30 s, sonicated in an ice bath for another 10 min, and centrifuged again under the aforementioned conditions. Finally, 120 μL of the clarified extract was transferred into a 2 mL LC/MS autosampler vial. To monitor system stability, 10 μL from each extracted sample was pooled to generate a quality control (QC) sample [29].

The liquid chromatography-mass spectrometry (LC-MS) system consisted of a Waters Acquity I-Class PLUS ultra-high-performance liquid chromatograph (Waters Corporation, Milford, MA, USA) coupled with a Waters Xevo G2-XS QTOF high-resolution mass spectrometer (Waters Corporation, Milford, MA, USA). Chromatographic separation was performed using an Acquity UPLC HSS T3 column (1.8 μm, 2.1 × 100 mm). Chromatographic separation was achieved utilizing a Waters Acquity UPLC HSS T3 column (1.8 μm, 2.1 × 100 mm). The mobile phase consisted of 0.1% aqueous formic acid (solvent A) and 0.1% formic acid in acetonitrile (solvent B), which were applied identically across both positive and negative electrospray ionization modes. The injection volume was set to 1 μL.

#### 2.2.2. LC-MS/MS Analysis

Primary and secondary mass spectrometry data were acquired in MSE mode using a Waters Xevo G2-XS QTOF high-resolution mass spectrometer (Waters Corporation, Milford, MA, USA) operated via MassLynx V4.2 software. Within a single acquisition cycle, concurrent dual-channel recording was executed at both low (2 V) and high (10–40 V) collision energies, with a scan time of 0.2 s per spectrum. The electrospray ionization (ESI) source was configured with capillary voltages of 2.0 kV for positive mode and 1.5 kV for negative mode, alongside a constant cone voltage of 30 V. Thermal conditions included a source temperature of 150 °C and a desolvation temperature of 500 °C, while the cone and desolvation gas flow rates were maintained at 50 L/h and 800 L/h, respectively [30].

#### 2.2.3. Qualitative and Quantitative Analysis of the Metabolites

Metabolic profiling was performed using a liquid chromatography-mass spectrometry (LC-MS) system, comprising a Waters Acquity I-Class PLUS ultra-performance liquid chromatograph (UPLC) coupled to a Waters Xevo G2-XS quadrupole time-of-flight (QTOF) high-resolution mass spectrometer. Chromatographic separation was achieved using an Acquity UPLC HSS T3 column (1.8 μm, 2.1 × 100 mm). Following data acquisition, subsequent data-processing operations, such as peak extraction, peak alignment, and normalization, were performed using Progenesis QI v3.0 software. Metabolite identification was based on the public METLIN database and Bemac’s self-constructed library, utilizing theoretical fragmentation identification. The deviation of the mass number of the parent ions was within 100 ppm, and the deviation of the fragment ion mass number was within 50 ppm [31]. The univariate analyses used were the *t*-test and fold-change analysis (FC analysis), while the multivariate analyses included principal component analysis (PCA) and partial least squares discriminant analysis (PLS-DA). The metabolite identification and pathway analysis were based on the KEGG database, and then, phytohormone and flavonoid metabolites in *L. discolor* were further identified by secondary mass spectrometry.

#### 2.2.4. Screening of the Differential Metabolites

A combination of univariate analysis and multivariate analysis, OPLS-DA (orthogonal partial least squares discriminant analysis), was used. Differentially expressed metabolites were identified based on the variable importance in projection (VIP ≥ 1) and univariate analysis q-values (q-value < 0.05) from the multivariate OPLS-DA model, combined with the multiplicity of the differences in the univariate analysis (fold-change ≥ 2 or ≤0.5), which were further screened to identify target metabolites with significant differences [32].

#### 2.2.5. Correlation Analysis of the Differential Metabolites

To elucidate the interplay between differentially accumulated phytohormones and metabolites in *L. discolor* across various light treatments, Pearson correlation coefficients were calculated based on their relative abundances. Subsequently, a phytohormone–secondary metabolite interaction network was generated utilizing Cytoscape software (v3.10.0), retaining only robust correlations that met strict threshold criteria (|r| > 0.9, *p* < 0.05) [33].

### 2.3. Transcriptomic Sequencing

#### 2.3.1. Sample Detection

Prior to transcriptome sequencing, RNA was extracted using the Trizol method, and the purity and concentration of the sample RNA were first detected using a spectrophotometer, NanoDrop 2000 (Thermo Fisher Scientific, Wilmington, DE, USA), and then, the integrity of the RNA was measured using an Agient 2100/LabChip GX Bioanalyzer (Agilent Technologies, Santa Clara, CA, USA) to ensure that the qualified samples were applicable for sequencing.

#### 2.3.2. Library Preparation

RNA-seq libraries were constructed starting with 1 μg of total RNA per sample, utilizing the NEBNext^®^ Ultra™ RNA Library Prep Kit for Illumina^®^ (NEB, Ipswich, MA, USA) according to the standard protocol. Briefly, poly-T oligo magnetic beads were applied to enrich mRNA, which was then fragmented at high temperatures using divalent cations. Reverse transcription into first-strand cDNA was mediated by random hexamers and M-MuLV Reverse Transcriptase, followed by second-strand synthesis using DNA Polymerase I and RNase H. The resulting fragments underwent end-repair to yield blunt ends, 3′-adenylation, and the ligation of NEBNext hairpin adaptors. Post-ligation, the AMPure XP system (Beckman Coulter, Brea, CA, USA) was employed to preferentially purify fragments around 240 bp. These size-selected fragments were digested with USER Enzyme (15 min at 37 °C, then 5 min at 95 °C) before undergoing PCR amplification with Phusion High-Fidelity DNA polymerase, universal, and index primers. The final library products underwent a secondary AMPure XP purification, and their integrity was assessed on an Agilent 2100 Bioanalyzer (Agilent Technologies, Santa Clara, CA, USA).

#### 2.3.3. Quality Control of Sequencing Data

The Illumina High-Throughput Sequencing Platform was used to sequence the cDNA libraries, producing a large amount of high-quality data (raw data), and the fastx toolkit (v0.0.14) was used to assess the sequencing-related quality of the raw sequencing data for each sample. High-quality clean data were obtained after removing reads containing junctions and removing low-quality reads (including reads with percentages of N greater than 10%, and reads with the number of bases with quality values of Q ≤ 10 accounting for more than 50% of the entire read).

#### 2.3.4. Sequence Assembly

Utilizing the Trinity v2.14.1 software, the assembly process commenced by dividing sequencing reads into k-mers. Through the analysis of sequence overlaps, these smaller k-mers were assembled into longer contigs and further clustered into distinct component collections. In the final step, transcript sequences were successfully derived from these collections by leveraging De Bruijn graph analysis combined with the underlying read data.

#### 2.3.5. Unigene Functional Annotation

Unigene sequences were aligned with the NR, Swiss-Prot, COG, KOG, eggNOG4.5, and KEGG databases using DIAMOND v2.1.8 [34] software, and KEGG Orthology results for Unigene in KEGG were obtained using KOBAS v3.0 [35]. InterProScan v5.61-93.0 [36] utilized the InterPro v95.0 integrated database to analyze the GO Orthology results of the new gene, and after predicting the amino acid sequence in Unigene, we used a HMMER v3.3.2 [37] database comparison to obtain the annotation information for Unigene.

#### 2.3.6. Mining of the Target Gene

To quantify gene expression levels, the sequencing reads were aligned against the assembled Unigenes utilizing Bowtie v2.4.5 [38]. Subsequently, the RSEM v1.3.3 tool [39] was employed to estimate transcript abundances, which were normalized as FPKM values. Furthermore, candidate genes involved in flavonoid biosynthesis were extracted from the transcriptome dataset based on KEGG pathway annotations. The FPKM metric [40] was computed according to the equation below:

$$FPKM=\frac{cDNA\;Fragments}{Mappedfragments(Millions)\;*\;TranscriptLength(kb)}$$

#### 2.3.7. Screening of the DEGs

The DEGs were screened based on the count value of the genes in each sample using DESeq2 v1.38.3 [41] software, and multiplicity of difference FC (fold-change) ≥ 2 and false discovery rate (FDR) < 0.01 were selected as the screening criteria.

#### 2.3.8. Correlation Analysis of Differential Phytohormones and DEGs

In order to further investigate the correlation between differential phytohormone levels and differential enzyme gene expression related to flavonoid metabolite biosynthesis pathways, data with |COR| > 0.8 and *p* < 0.05 were selected for correlation analysis. The corresponding “phytohormone–enzyme gene expression” correlation network was constructed using Cytoscape 3.10.0 software.

#### 2.3.9. Regulatory Network of DEGs and Differential Metabolites

Following the methodology described by Ji [42], differentially expressed genes (DEGs) and differential metabolites were co-mapped onto targeted KEGG pathways. Subsequently, Pearson correlation analysis was conducted via IBM SPSS Statistics 21 to evaluate the relationship between transcript expression levels and relative metabolite abundances, applying strict filtering criteria (|r| > 0.9, *p* < 0.05).

#### 2.3.10. Transcription Factor Prediction

To identify putative transcription factors (TFs) across the *L. discolor* transcriptome, all gene sequences were queried against the PlantTFDB database (http://planttfdb.gao-lab.org/, accessed on 24 February 2024). The resulting TF families were subsequently cataloged and statistically analyzed using Microsoft Excel. To elucidate the regulatory dynamics between these TFs and biosynthetic enzyme genes, Pearson correlation coefficients were calculated. Significant co-expression pairs (|r| > 0.90, *p* < 0.05) were then visualized as a putative TF–enzyme regulatory network utilizing Cytoscape (v3.10.0).

#### 2.3.11. Analysis of the Cis-Element Components of the Transcription Factor Promoters

In order to analyze the possible regulatory mechanisms of transcription factors related to flavonoid biosynthesis in *L. discolor* in response to phytohormones, the transcription factors were analyzed using the Nation Center for Biotechnology Information (NCBI; http://www.ncbi.nlm.nih.gov, accessed on 24 February 2024) website. The nucleic acid sequences of 2000 bp upstream of the transcription start sites of the transcription factors were obtained, and the sequences were imported into PlantCARE (http://bioinformatics.psb.ugent.be/webtools/plantcare/html/, accessed on 24 February 2024) for promoter cis-element analysis; finally, the transcripts were visualized using the TBtools 2.102 for visual mapping.

#### 2.3.12. qRT-PCR Analysis

To further verify the reliability of the results for DEGs, DEGs under light quality treatment were selected and qRT-PCR was applied to verify the gene expression and changes. Amplification conditions: 95 °C for 30 s and then 95 °C for 10 s and 63 °C for 30 s, with 40 cycles. Melting curve procedures used to determine the reaction specificity: 95 °C for 15 s, 60 °C for 60 s, and 95 °C for 15 s. The relative expression level of target genes was calculated using the 2^−ΔΔCt^ method. At least three independent biological replicates were set up for each gene in all qRT-PCR assays. Statistical analysis of the expression data was performed using GraphPad Prism 10.6 software. One-way ANOVA was used to evaluate the overall differences among treatment groups, followed by Tukey’s post hoc test for pairwise multiple comparisons, with a statistically significant difference defined as *p* < 0.05. The primers were designed using Primer 5 software, and the primers for the internal reference gene *26SrRNA* and target gene are shown in Table 1.

## 3. Results

### 3.1. Metabolomics Analysis

#### 3.1.1. Repeated Correlation Assessment and PCA

**Figure 1:**
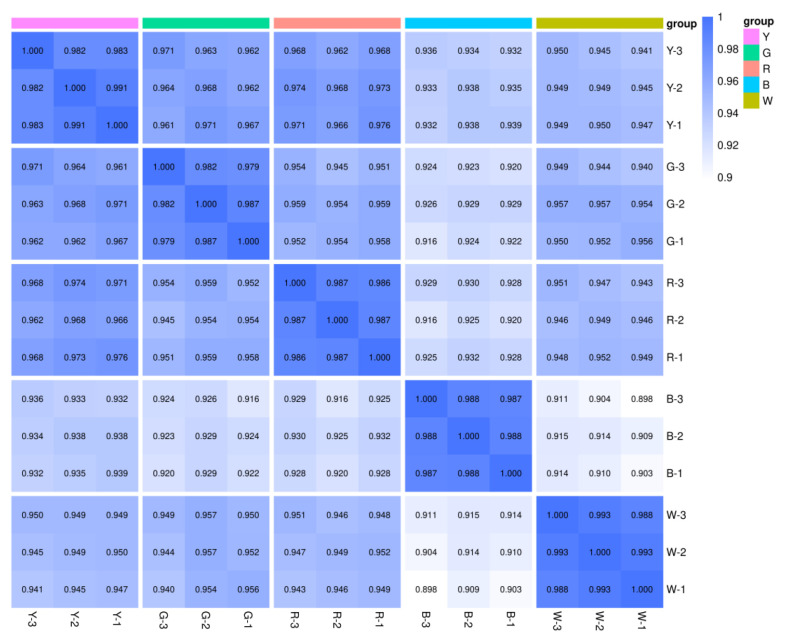
Repeat correlation assessment. Note: The abscissa represents the sample name, the ordinate represents the corresponding sample name; the color represents the size of the correlation coefficient value. W, white light; B, blue light; R, red light; G, green light; Y, yellow light. W-1, W-2, and W-3 represent three replicates of W; B-1, B-2, and B-3 represent three replicates of B; R-1, R-2, and R-3 represent three replicates of R; G-1, G-2, and G-3 represent three replicates of G; Y-1, Y-2, and Y-3 represent three replicates of Y. The same applies below.

The Spearman rank correlation (r) was closer to 1, indicating that the two replicate samples were more strongly correlated. As illustrated in Figure 1, correlation between intra-group biological replicates was remarkably high.

**Figure 2:**
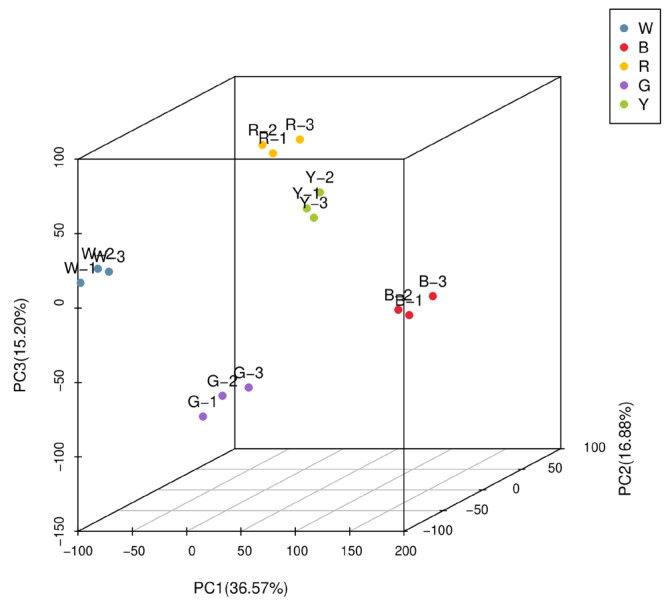
Sample principal component analysis. Note: PCA score map of the mass spectrometry data of all the samples and quality control samples, where the X-axis represents the first principal component, the Y-axis represents the second principal component, and the z-axis represents the third principal component.

The principal component analysis (PCA) results are shown in Figure 2. The results for PC1, PC2, and PC3 were 36.57%, 16.88%, and 15.2%, respectively, with a clear metabolite group separation between the five treatments indicating that the sample metabolites differed among the groups. Meanwhile, the PCA showed minimal variability among biological replicates, which indicated a good correlation between the sample replicates.

#### 3.1.2. Analysis of Phytohormone Differential Metabolites

In order to analyze the distribution of the metabolic differences in phytohormones and important active metabolites in *L. discolor* among different treatments, the present study further screened phytohormones and flavonoid metabolites with significant differences by using VIP ≥ 1, q-value < 0.05, and fold-change ≥ 1 as the screening conditions.

**Figure 3:**
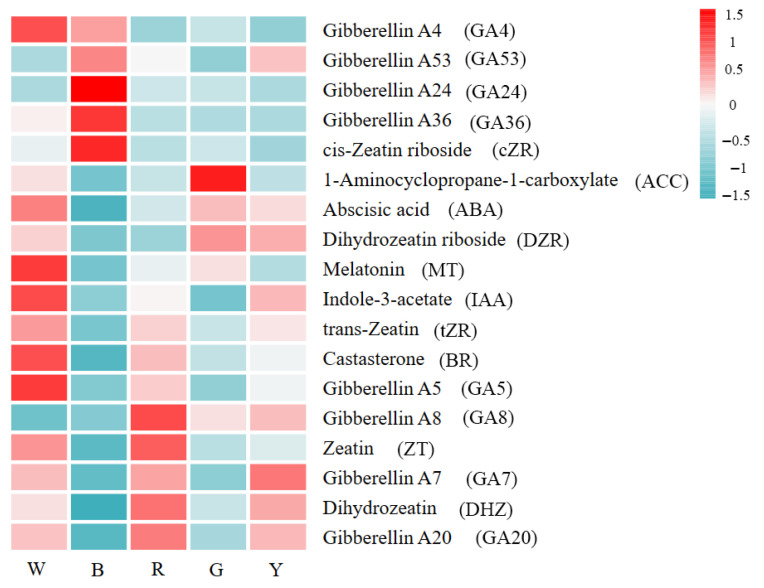
Heatmap of differential phytohormone metabolites in leaves of *L. discolor* under different light quality treatments.

*L. discolor* leaves were screened for a total of 18 differential phytohormones in response to five different light quality treatments, as shown in Figure 3. W_vs_B had 14 differential phytohormones, including 13 down-regulated metabolites; W_vs_R had 12 differential phytohormones, with 5 up-regulated and 7 down-regulated; W_vs_G had 11 differential phytohormones, with 3 up-regulated and 8 down-regulated; and W_vs_Y had 10 differential phytohormones, with 3 up-regulated and 7 down-regulated. Treatment with blue light led to the fewest differentially expressed phytohormones but the highest number of down-regulated phytohormones and the lowest total relative content of differential phytohormones. The highest number of up-regulated differential phytohormones and the highest total relative content of phytohormones were found under red light illumination. It was hypothesized that red light illumination conditions were favorable for the accumulation of *L. discolor* phytohormone metabolites. In terms of the biosynthesis of gibberellin (GA), a total of eight differential metabolites were found. Among them, G24, G53, and G36 had the highest relative contents under blue light, accounting for 54.66%, 34.13%, and 29.38%, respectively; G8 and G20 had the highest relative contents under red light, accounting for 27.40% and 26.04%, respectively; G4 and G5 had the highest relative contents in white light, accounting for 25.16% and 26.48%. In terms of zeatin (ZT) synthesis, five differential metabolites were identified; ZT and DZT had the highest relative contents under red light, accounting for 27.87% and 22.84%, respectively; and DZR, tZT, and cZR had the highest relative contents under green (22.32%), white (23.30%), and blue (57.41%) light. IAA, MT, ABA, and BR had the highest relative content under white light, accounting for 22.36%, 33.38%, 21.57%, and 25.30%, respectively. Among the ethylene biosynthesis pathways, ACC had the highest relative content under green light. Specific changes in phytohormones are shown in Supplementary Table S1.

#### 3.1.3. Analysis of the Differential Flavonoid Metabolites

**Figure 4:**
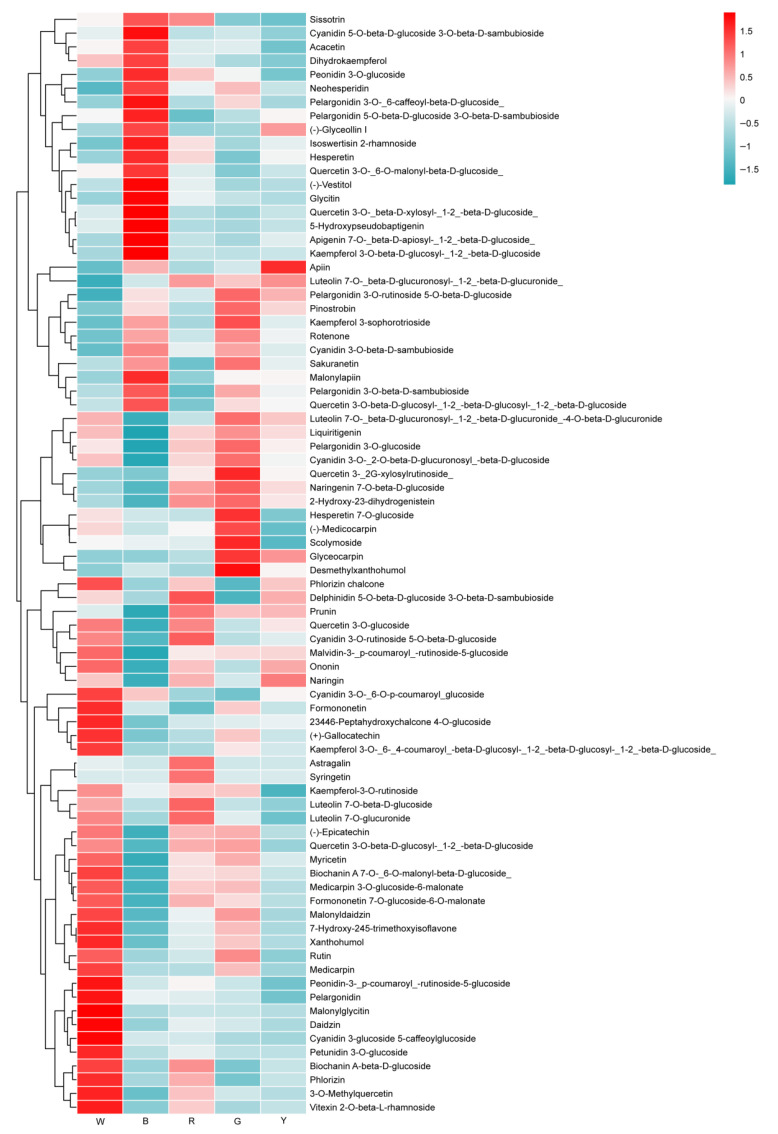
Heatmap of different flavonoid metabolites in leaves of *L. discolor* under different light quality treatments.

As shown in Figure 4, a total of 80 flavonoid metabolites differed significantly in *L. discolor* leaves under different light quality treatments. Among the flavanone metabolites, Pinostrobin, Neohesperidin, and Hesperetin were enriched under blue light; Naringenin 7-O-beta-D-glucoside and Prunin were enriched under red light; Hesperetin 7-O-glucoside, (Neohesperidi), Naringenin 7-O-beta-D-glucoside, and Sakuranetin were enriched in yellow light; and Neohesperidin was enriched in green light. Among the chalcone and dihydrochalcone metabolites, Desmethylxanthohumol was enriched in blue, yellow, and green lights. Among the flavonoids and flavonol metabolites, Kaempferol 3-O-beta-D-glucosyl-(1->2)-beta-D-glucoside, Apigenin 7-O-[beta-D-apiosyl-(1->2)-beta-D-glucoside], Quercetin 3-O-[beta-D-xylosyl-(1->2)-beta-D-glucoside], and 10 other metabolites were enriched under blue light; Apigenin 7-O-[beta-D-apiosyl-(1->2)-beta-D-glucoside], Peonidin 3-O-glucoside, and five other metabolites were enriched in red light; six metabolites, including Quercetin 3-(2G-xylosylrutinoside) and Scolymoside, were enriched in green light; and Kaempferol 3-O-beta-D-glucosyl-(1->2)-beta-D-glucoside, Quercetin 3-(2G-xylosylrutinoside), and seven other metabolites were enriched under yellow light. Among the isoflavonoid metabolites, five metabolites, including Glycitin and (-)-Vestitol, were enriched under blue light; three metabolites, including Glycitin, were enriched under red light; four metabolites, including Glyceocarpin and Rotenone, were enriched under green light; and four metabolites, including Glyceocarpin and (−)-Glyceollin I, were enriched under yellow light. Among the anthocyanin metabolites, six metabolites, including Cyanidin 5-O-beta-D-glucoside 3-O-beta-D-sambubioside and Pelargonidin 3-O-beta-D-sambubioside, were enriched under blue light; Pelargonidin 3-O-beta-D-sambubioside, Peonidin 3-O-glucoside, and three other metabolites were enriched under red light; Cyanidin 3-O-beta-D-sambubioside, Cyanidin 3-O-(2-O-beta-D-glucuronosyl)-beta-D-glucoside, and five other metabolites were enriched under green light; and the only metabolite enriched under yellow light was Cyanidin 3-O-rutinoside 5-O-beta-D-glucoside. The specific changes in flavonoid metabolites are shown in Supplementary Table S2.

Overall, the contents of the flavonoid metabolites of *L. discolor* under blue light treatment were higher than under the other light treatments, and Pinostrobin, Hesperetin, Malonylapiin, Quercetin 3-O-(6-O-malonyl-beta-D-glucoside), Quercetin 3-O-beta-D-glucosyl-(1->2)-beta-D-glucosyl-(1->2)-beta-D-glucoside, (-)-Vestitol, 5-Hydroxypseudobaptigenin, Pelargonidin 3-O-beta-D-sambubioside, and other metabolites may be the main substances responsible for the increase.

#### 3.1.4. Correlation Analysis Between Differential Phytohormones and Flavonoid Metabolites

**Figure 5:**
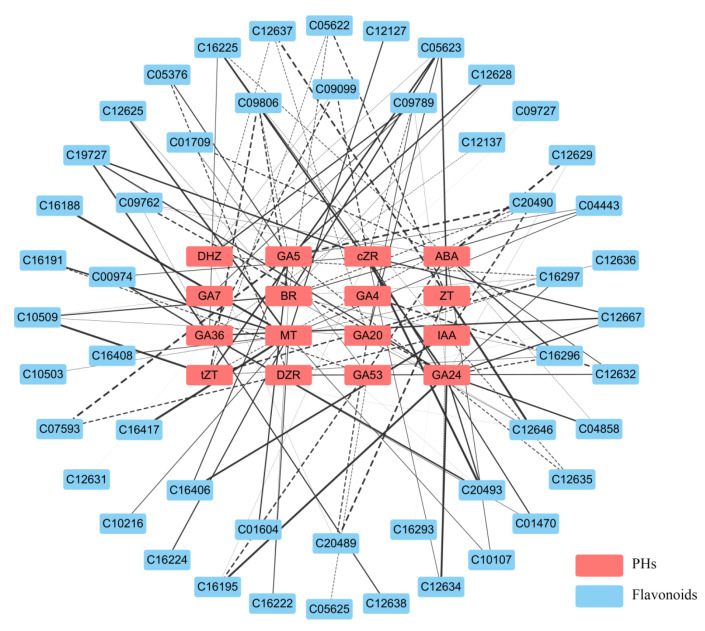
Correlation network diagram for “phytohormone–flavonoid metabolites” in the leaves of *L. discolor.* Note: Flavonoid metabolites are represented by KO numbers, and the specific phytohormone and flavonoid metabolites are named in Supplementary Table S3. The red boxes indicate differential phytohormone metabolites (PHs), the blue boxes indicate differential flavonoid metabolites (flavonoids), the solid lines indicate positive correlations, and the dashed lines indicate negative correlations.

To further investigate the relationship between differential phytohormones and flavonoid-related differential metabolites in *L. discolor* leaves under different light quality treatments, the present study used Pearson correlation analysis (|COR| > 0.9, *p* < 0.05) and construction of a “phytohormone–active metabolite” correlation network diagram. As shown in Figure 5, the network comprised 16 phytohormones and 50 flavonoids with significant correlations. These metabolites included 16 flavones/flavonols, 14 isoflavonoids, 8 anthocyanins, 4 flavanones, 4 chalcones/dihydrochalcones, and 2 flavanols. Among them, 16 were flavonoids or flavonols, 14 were isoflavonoids, 8 were anthocyanins, 4 were flavanones, 4 were chalcones or dihydrochalcones, and 2 were flavanols. Among the gibberellins, GA24 showed a strong positive correlation with nine metabolites, including (-)-Vestitol and Cyanidin 5-O-beta-D-glucoside 3-O-beta-D-sambubioside; GA36 showed a strong positive correlation with Quercetin 3-O-[beta-D-xylosyl-(1->2)-beta-D-glucoside], Cyanidin 5-O-beta-D-glucoside 3-O-beta-D-sambubioside, and seven other flavonoid metabolites, but not with 2-Hydroxy-2,3-dihydrogenistein or Liquiritigenin; GA53 was strongly positively correlated with Hesperetin; GA5 was strongly positively correlated with Biochanin A-beta-D-glucoside, Vitexin 2″-O-beta-L-rhamnoside, and Neohesperidin; and GA20 showed strong negative correlation with Quercetin 3-O-beta-D-glucosyl-(1->2)-beta-D-glucosyl-(1->2)-beta-D-glucoside and Pelargonidin 3-O-(6-caffeoyl-beta-D-glucoside). MT showed strong positive correlations with (+)-Gallocatechin, Xanthohumol, 7-Hydroxy-2′,4′,5′-trimethoxyisoflavone, and Malonyldaidzin, and IAA showed strong positive correlations with Neohesperidin and Rotenone. ABA had a strong positive correlation with Myricetin and a strong negative correlation with Isoswertisin 2″-rhamnoside and (-)-Vestitol. BR had a strong negative correlation with Quercetin 3-O-glucoside and 3-O-Methylquercetin, and a strong positive correlation with Cyanidin 3-O-beta-D-sambubioside. Among the zeatin-like phytohormones, DHZ showed strong positive correlations with Naringin and Quercetin 3-O-glucoside, a strong negative correlation with Pelargonidin 3-O-(6-caffeoyl-beta-D-glucoside), strong positive correlations with tZT and Ononin, and a strong positive correlation with Pelargonidin 3-O-(6-caffeoyl-beta-D-glucoside); tZT had a strong positive correlation with Quercetin 3-O-glucoside and Pelargonidin 3-O-(6-caffeoyl-beta-D-glucoside); and there were strong positive correlations between cZR and flavonoid metabolites such as (-)-Vestitol and Cyanidin 5-O-beta-D-glucoside 3-O-beta-D-sambubioside. The correlations of differential phytohormones and differential flavonoid metabolites are shown in Supplementary Table S3.

### 3.2. Transcriptomic Analysis

#### 3.2.1. Library Construction

Through the use of Illumina HiSeq 2000 high-throughput sequencing to complete the transcriptome sequencing of 15 samples, a total of 96.89 Gb of clean data were obtained in this experiment; the clean data per sample reached 5.81 Gb, with the percentage of Q30 bases at 92.41% and above, and the sequencing accuracy was high. After assembly, a total of 69,897 Unigenes were obtained conducted, 22,987 of which were longer than 1 kb; their N50 length was 2428 bp and their GC content was greater than 46.50%, which indicated that the stability of the instrument was good during the sequencing process, the quality of the constructed gene libraries was high, and they could be used for the subsequent analysis (the results are shown in Supplementary Tables S4 and S5).

#### 3.2.2. DEG Analysis

**Figure 6:**
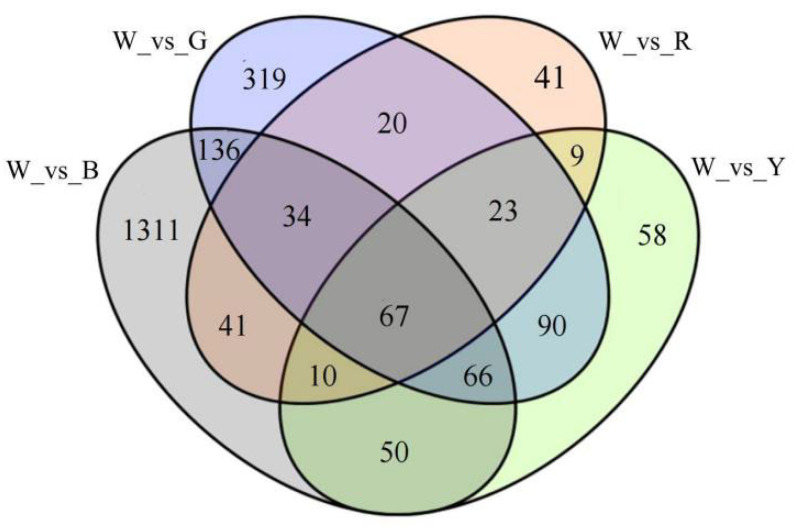
Venn diagram of differentially expressed genes in the leaves of *L. discolor* under different light quality treatments. Note: W_vs_B, white light compared to blue light; W_vs_G, white light compared to green light; W_vs_R, comparison of white light with red light; W_vs_Y, white light compared to yellow light.

DEGs were screened based on the gene expression in individual samples (with fold-change ≥ 2 and FDR < 0.01 as the screening criteria), and a Venn diagram was used to represent the DEGs between samples (Figure 6). A total of 1715 DEGs (1059 down-regulated and 656 up-regulated) were found in W vs. B; 755 DEGs (380 down- and 375 up-regulated) were found in W vs. G; 2455 DEGs (91 down- and 154 up-regulated) were found in W vs. R; and 375 DEGs (120 down- and 253 up-regulated) were found in W vs. Y. In these comparisons, there were 1131, 319, 41, and 58 DEGs in the comparison groups of W_vs_B, W_vs_G, W_vs_R, and W_vs_Y, respectively, suggesting that these genes were involved in the plant’s response to light under different light quality treatments. *L. discolor* leaves were more sensitive to blue light and responded more positively to it than to red, yellow, and green light.

#### 3.2.3. Analysis of the Genes Involved in the Biosynthesis of the Flavonoid Metabolites

Genes related to flavonoid biosynthesis and metabolism in *L. discolor* were further screened based on KEGG pathway enrichment, and 87 genes involved in flavonoid biosynthesis were selected in this study, as shown in Supplementary Table S6. Among them were 20 genes in the phenylpropane metabolism (No.Ko00940) pathway, including *4CL*, *PAL*, and *CYP73A*; 57 genes in the flavonoid biosynthesis (No.Ko00941) pathway, including *HCT*, *CHS*, *CHI*, *CCoAOMT*, *CYP73A*, *C12RT1*, *PGT1*, *F3′H*, *FLS*, *ANR*, *F3′5′H*, *CYP98A*, and *F3H*; 1 gene in the anthocyanidin biosynthesis (No.Ko00942) pathway, *3AT*; 11 genes in the isoflavone biosynthesis (No.Ko00943) pathway, including *CYP81E*, *IF7MAT*, and *CYP71D9*; and 14 genes in the flavonoid and flavonol biosynthesis (No.Ko00944) pathway, including *C12RT1*, *IF7MAT*, *F3′H*, *FG2*, *F3′5′H*, and *UGT73C6*.

#### 3.2.4. Analysis of DEGs Associated with Biosynthesis of Flavonoid Compounds

**Figure 7:**
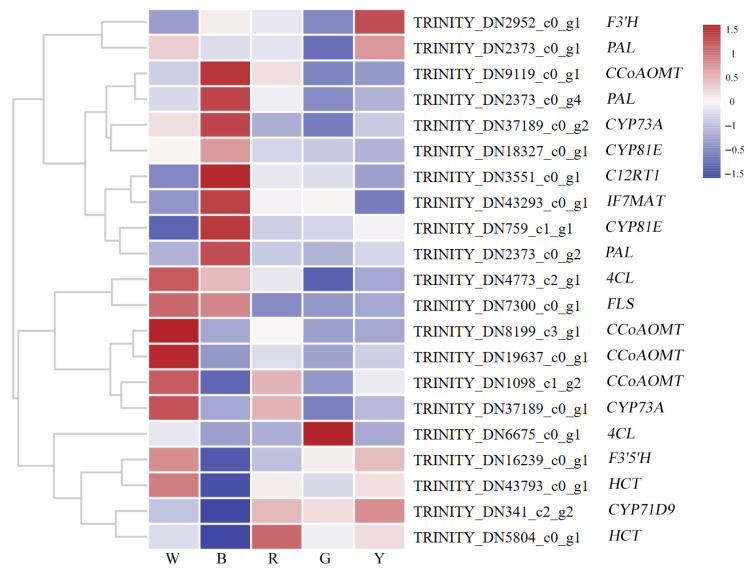
Cluster heatmap of DEGs related to flavonoid biosynthesis in the leaves of *L. discolor* under different light quality treatments.

A total of 21 DEGs related to flavonoid biosynthesis pathway were further screened out, and the cluster analysis of the DEGs was performed using the FPKM values (see Figure 7). There were six up-regulated genes and nine down-regulated genes in W_vs_B. The up-regulated genes encoded enzymes including *C12RT1*, *F3′H*, *IF7MAT*, *PAL*, *CCoAOMT*, and *CYP81E*; the down-regulated genes involved enzymes including *CCoAOMT*, *CYP73A*, *F3′5′H*, *HCT*, *4CL*, and *CYP71D9*. Three differentially expressed genes of *CCoAOMT* (TRINITY_DN1098_c1_g2, TRINITY_DN19637_c0_g1, and TRINITY_DN8199_c3_g1) were significantly down-regulated. In W_vs_G, there was one up-regulated gene and there were nine down-regulated genes. The up-regulated gene was *4CL* (TRINITY_DN6675_c0_g1); the down-regulated genes included *CYP73A*, *CCoAOMT*, *PAL*, *4CL*, and *FLS*. In W_vs_R, there was one up-regulated gene and two down-regulated genes. The up-regulated gene was the enzyme *F3′H* (TRINITY_DN2952_c0_g1); the enzymes in the down-regulated genes included *FLS* and *CCoAOMT*. In W_vs_G, there was one up-regulated gene and five down-regulated genes, the up-regulated enzyme-encoding gene was, again, *F3′H* (TRINITY_DN6675_c0_g1); meanwhile, the down-regulated enzyme-encoding genes included *CYP73A*, *CCoAOMT*, *FLS*, and *CYP81E*. Further details regarding the flavonoid DEGs are shown in Supplementary Table S7.

#### 3.2.5. Correlation Analysis of DEGs and Differential Phytohormone and Flavonoid Metabolic Components

**Figure 8:**
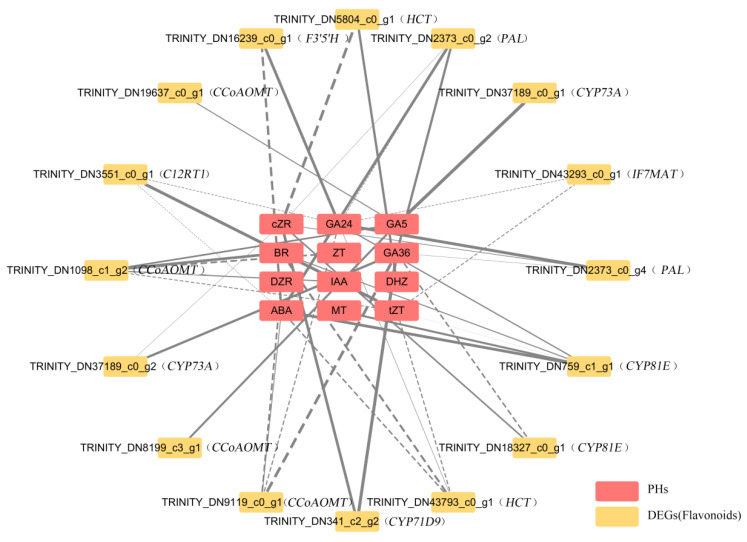
Correlation network of “phytohormone–flavonoid differentially expressed genes” in the leaves of *L. discolor.* Note: The red boxes indicate differential endogenous phytohormone metabolites (PHs), and the orange boxes indicate the flavonoid synthetase differentially expressed genes (DEGs). The solid lines indicate positive correlations, the dashed lines indicate negative correlations, and the thicker the line, the stronger the correlation.

In order to further investigate the correlations between differential phytohormone and flavonoid metabolite and enzyme-encoding differentially expressed genes (DEGs) in *L. discolor* leaves under different light quality conditions, Pearson’s correlation analysis was performed (|COR| > 0.8, *p* < 0.05) and a correlation network diagram was constructed. The correlation network diagram of “Phytohormone–Flavonoid DEGs” was constructed. As shown in Figure 8, a total of 16 DEGs for 6 phytohormones (IAA, GA, MT, ZT, ABA, and BR) and 9 key enzymes for flavonoid biosynthesis (*PAL*, *HCT*, *CYP73A*, *F3′5′H*, *CCoAOMT*, *CYP81E*, *IF7MAT*, *C12RT1*, and *CYP71D9*) were correlated with each other. TRINITY_DN2373_c0_g4 and TRINITY_DN2373_c0_g2 had positive correlations with GA24, GA36, and cZR, but *PAL* (TRINITY_DN2373_c0_g2) had a negative correlation with ABA; *HCT* had positive correlations with GA24, cZR, DHZ, and tZT, but *HCT* (TRINITY_DN43793_c0_g1) was negatively correlated with ABA and BR; *CYP73A* was positively correlated with GA36, GA5, and cZR; *F3′5′H* was positively correlated with ABA but negatively correlated with GA24; *CCoAOMT* was positively correlated with IAA, GA24, GA36, GA5, tZT, ZT, and BR, but *CCoAOMT* (TRINITY_DN9119_c0_g1) was negatively correlated with DZR and ABA; *CYP81E* was positively correlated with GA24, GA36, and cZR, but *CYP81E* (TRINITY_DN759_c1_g1) was negatively correlated with MT; *IF7MAT* had a positive correlation with GA24 and negative correlation with tZT; *C12RT1* had a positive correlation with GA24 only, but negative correlations with tZT and ABA; and *CYP71D9* had negative correlations with GA36 and cZR. The specific results are shown in Supplementary Table S8.

#### 3.2.6. Integrated Transcriptome and Metabolome Analysis of the Flavonoid Compound Accumulation Regulatory Network

**Figure 9:**
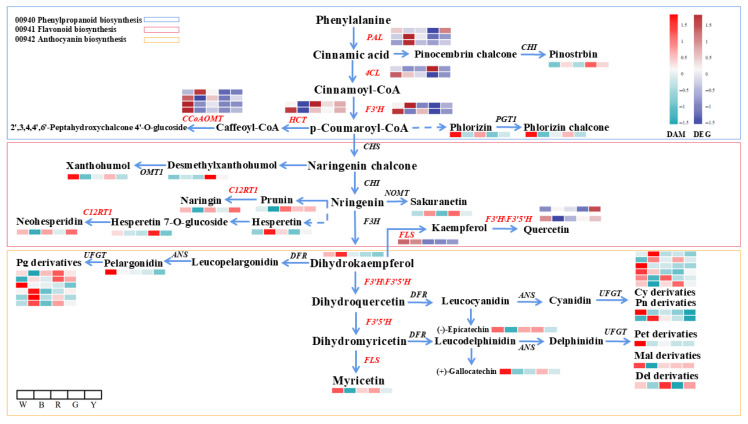
Regulatory network of DEGs and differentially expressed metabolites in *L. discolor* leaves under different light quality treatments. Note: The heatmap from left to right shows the W, B, R, G, and Y treatments, representing the gene expression levels (DEGs) and relative metabolite content levels (DAMs) of *L. discolor* leaves under different light treatments.

To elucidate the regulatory relationship between flavonoid-related metabolites and differentially expressed genes (DEGs) in *L. discolor* leaves, Spearman correlation coefficients were calculated. Stringent thresholds of |r| > 0.9 and *p* < 0.05 were applied to identify robust correlations, the details of which are provided in Supplementary Table S9. Furthermore, these differential components were co-mapped onto the flavonoid biosynthetic pathway, as illustrated in Figure 9. The joint metabolome and transcriptome analysis showed that differential genes in the metabolic pathway of flavonoid compounds were closely related to the synthesis of the differential metabolites, including flavonoids, flavonols, isoflavones, and anthocyanins. The results revealed a positive correlation between the *PALs* gene and Hesperetin, Dihydrokaempferol, Cyanidin 5-O-beta-D-glucoside 3-O-beta-D-sambubioside, (-)-Epicatechin, Malvidin-3-(*p*-coumaroyl)-rutinoside-5-glucoside, and Pelargonidin 3-O-glucoside; *4CL* was positively correlated with Hesperetin 7-O-glucoside and *CYP73A* with Dihydrokaempferol, Phlorizin, and Pelargonidin 5-O-beta-D-glucoside 3-O-beta-D-sambubioside; *CYP73A* was positively correlated with Naringenin 7-O-beta-D-glucoside, Pelargonidin 3-O-glucoside, and others; *F3′5′H* was positively correlated with Malvidin-3-(p-coumaroyl)-rutinoside-5-glucoside and negatively correlated with Peonidin 3-O-glucoside; *HCT* was positively correlated with Malvidin-3-(p-coumaroyl)-rutinoside-5-glucoside and negatively correlated with Peonidin 3-O-glucoside; *FLS* was negatively correlated with Naringenin 7-O-beta-D-glucoside. *CYP81E* was positively correlated with Neohesperidin and Pelargonidin 3-O-(6-caffeoyl-beta-D-glucoside) and negatively correlated with Myricetin, Malvidin-3-(p-coumaroyl)-rutinoside-5-glucoside, and (-)-Epicatechin. *IF7MAT* was negatively correlated with Neohesperidin, Peonidin-3-(p-coumaroyl)-rutinoside-5-glucoside, Pelargonidin 3-O-(6-caffeoyl-beta-D-glucoside), etc. There was also a positive correlation. This result further suggests that the regulation of flavonoid compounds in *L. discolor* consists of multiple gene interactions.

#### 3.2.7. Prediction Analysis of Transcription Factors and Metabolic Enzyme Genes Using the Transcription Factor Analysis Tool in the PlantTFDB Website

A total of 422 genes were predicted to be involved in encoding 47 transcription factor families according to the *L. discolor* transcriptome data under the different light quality treatments. Among them, the top 10 transcription factor families were C2H2 (34), bZIP (27), bHLH (27), MYB_related (27), GRAS (25), ERF (24), NAC (20), FAR1 (20), and MYB (18).

**Figure 10:**
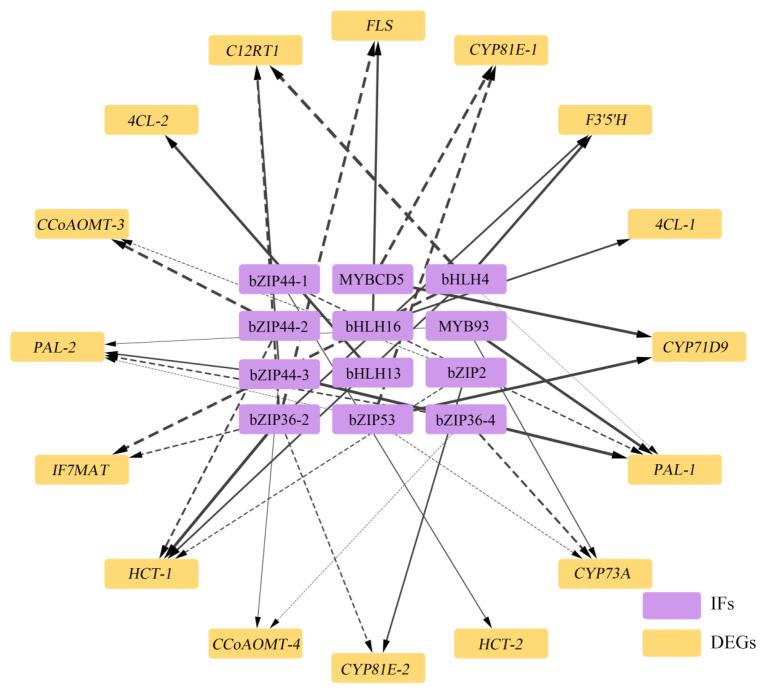
Regulatory network diagram of “transcription factor–flavonoid differential expressed genes” in the leaves of *L. discolor.* Note: The purple boxes indicate the transcription factors (TFs), the orange boxes indicate the differential enzyme genes of the flavonoid biosynthesis pathway (DEGs); the directions of the arrows indicate the directions of regulation, the solid lines indicate positive regulation, the dotted lines indicate negative regulation, and the thicker the line, the stronger the regulatory correlation.

In order to further analyze the key transcription factors involved in the regulation of flavonoid biosynthesis in *L. discolor*, Pearson correlation analysis was performed to filter out the data with correlation coefficients greater than 90% between the transcription factors and the enzyme genes, and the regulation of the three transcription factor families selected, namely, MYB, bHLH, and bZIP, was predicted in conjunction with the PlantTFDB website; a regulatory network diagram of “transcription factor–enzyme gene expression” was constructed. We also constructed a “transcription factor–enzyme gene expression” regulatory network diagram. As shown in Figure 10, there was a regulatory relationship between 10 transcription factors, including MYBCD5, MYB88, bHLH16, bHLH13, and bZIP53, and 16 flavonoid biosynthesis differential enzyme genes for 11 enzymes, including *PAL*, *HCT*, *FLS*, *4CL*, *F3′5′H*, *FLS*, and *CYP73A*.

Among them, MYBCD5 negatively regulated *CYP81E-1* and positively regulated *CYP71D9*; bHLH13 positively regulated *4CL-2*; bHLH4 positively regulated *F3′5′H* and *HCT-1*, and negatively regulated *PAL-1*, *C12RT1*, and *IF7MAT*; bHLH16 positively regulated *FLS* and *4CL-1*; MYB93 positively regulated *PAL-2*, *PAL-3*, and *CYP73A*; bZIP53 positively regulated *CYP71D9* and negatively regulated *CYP81E-1*, *PAL-2*, and *CYP73A*; bZIP44 positively regulated *HCT-2*, *PAL-2*, *C12RT1-1*, and *CCoAOMT-4*, and negatively regulated *HCT-1* and *CCoAOMT-3*; bZIP36 positively regulated *F3′5′H* and *HCT-1*, and negatively regulated *C12RT1*, *IF7MAT*, *CYP81E-2*, *PAL-2*, *CYP73A*, and *CCoAOMT-4*; bZIP2 positively regulated *CYP81E-2* and negatively regulated *HCT-1* and *CCoAOMT-3*; MYB88 positively regulated *FLS*; and bHLH positively regulated *F3′5′H* and *HCT-1*, but negatively regulated *C12RT1* and *IF7MAT*. The co-expression of flavonoid-related transcription factors and enzyme genes is shown in Supplementary Table S10.

#### 3.2.8. Analysis of the Mechanism by Which Flavonoid Synthesis Is Regulated by the Differential Accumulation of Phytohormones

**Figure 11:**
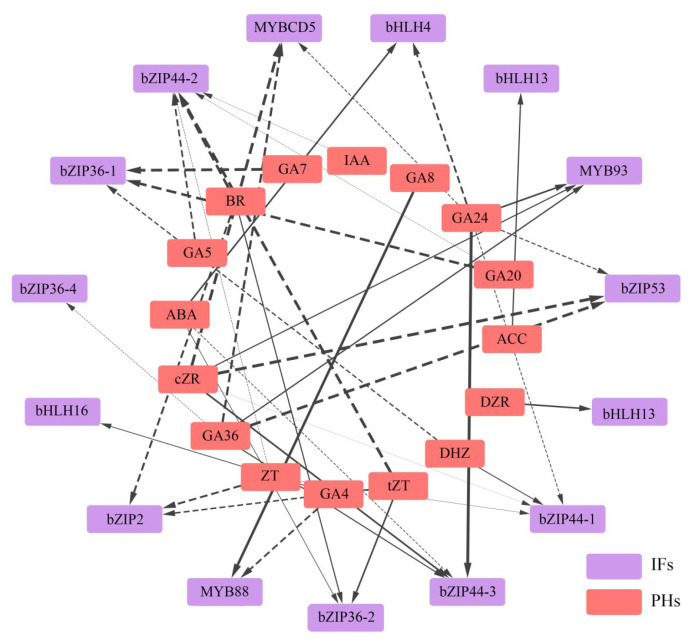
Regulatory network map of “phytohormone–transcription factor” in the leaves of *L. discolor.* Note: The purple boxes indicate the transcription factor, the red boxes indicate differential endogenous phytohormone metabolites, the directions of the arrows indicate the directions of regulation, the solid lines indicate positive regulation, the dotted lines indicate negative regulation, and the thicker the line, the stronger the regulatory correlation.

The relationship between phytohormones and transcription factors in *L. discolor* leaves under different light quality treatments was further explored, and the relationship between phytohormone content and transcription factor expression was determined using Pearson correlation coefficients (Figure 11). IAA had a significant negative correlation with bZIP44; GA24 had a negative correlation with MYBCD5, bHLH4, bZIP53, and bZIP44 (TRINITY_DN1743_c1_g1), but was positively correlated with MYB93; GA4 was negatively correlated with MYB88 and positively correlated with BHLH16; GA36 was negatively correlated with MYBCD5, bZIP53, and bZIP36 (TRINITY_DN9_c0_g2); ACC was positively correlated with bHLH13; ZT was negatively correlated with bZIP2 and bZIP44 (TRINITY_DN1743_c1_g2); and ABA was positively correlated with bHLH4 and bZIP36 (TRINITY_DN4112_c0_g1), but negatively correlated with bZIP44 (TRINITY_DN1743_c2_g1). The specific correlation coefficients are shown in Supplementary Table S11.

To further analyze the possible regulatory mechanisms of key transcription factors related to flavonoid biosynthesis in the phytohormone responses of *L. discolor* leaves under different light quality treatments, promoter cis-element analyses were performed for each of the predicted transcription factors. These included an auxin-responsive element (TGA-element), cis-acting regulatory element involved in auxin responsiveness (AuxRR-core), gibberellin-responsive element (P-box/GARE-motif), cis-acting element involved in gibberellin-responsiveness (TATC-box), cis-acting regulatory element involved in MeJA-responsiveness (TGACG-motif/CGTCA-motif), and cis-acting element involved in abscisic acid responsiveness (ABRE). Details regarding the promoter elements of transcription factors associated with flavonoid biosynthesis in *L. discolor* leaves are provided in Supplementary Table S12, categorized by different light quality treatments.

**Figure 12:**
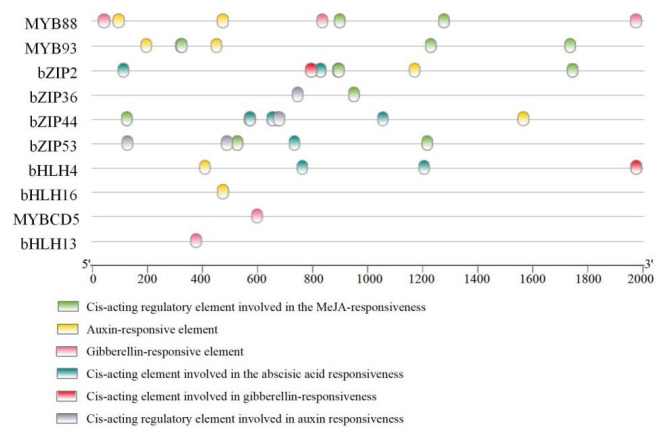
Promoter cis-element analysis for flavonoid metabolites and related transcription factors in the leaves of *L. discolor*.

As illustrated in Figure 12, among the 10 transcription factors regulating flavonoid biosynthetic enzymes, MYBCD5 and bHLH13 responded to GA phytohormone regulation; bHLH16 responded to AUX phytohormone regulation; MYB93 and bZIP36 responded to two phytohormones, Me_JA and AUX; MYB88 responded to three phytohormones, Me_JA, AUX, and GA; bHLH4 responded to three phytohormones, ABA, AUX, and GA; ZIP53 and bZIP44 responded to three phytohormones, Me_JA, AUX, and ABA; and bZIP2 responded to four phytohormones, Me_JA, ABA, GA, and AUX.

#### 3.2.9. qRT-PCR Validation of Key Transcription Factors and Enzyme Genes

**Figure 13:**
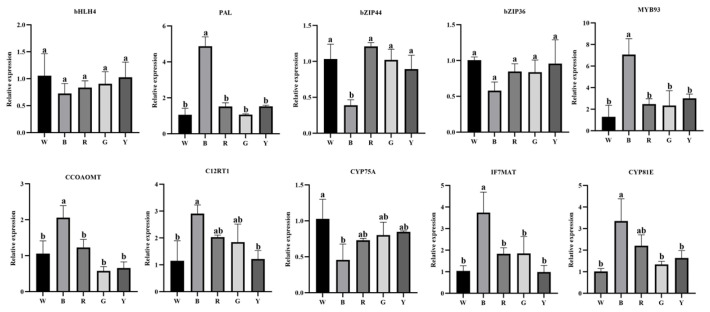
qRT-PCR analysis of differential enzymes and transcription factors in the leaves of *L. discolor* under different light treatments. Different lowercase letters indicate significant differences among different treatment groups at *p* < 0.05 (one-way ANOVA followed by Tukey’s post hoc test).

To verify the expression profiles of essential transcription factors and biosynthetic enzymes involved in the flavonoid pathway of *L. discolor* under varied light qualities, ten representative genes were subjected to qRT-PCR analysis. These candidates were prioritized from the previously identified key regulatory and structural genes. As shown in Figure 13, the enzyme genes *C12RT1* (TRINITY_DN3551_c0_g1), *CCoAOMT* (TRINITY_DN9119_c0_g1), *PAL* (TRINITY_DN2373_c0_g2), *CYP81E* (TRINITY_DN759_c1_g1), and *IF7MAT* (TRINITY_DN43293_c0_g1) and the transcription factor MYB93 (TRINITY_DN6460_c0_g1) were significantly higher under blue light than in the control W. The expression of the enzyme gene *F3′5′H* (TRINITY_DN16239_c0_g1) and transcription factors bZIP44 (TRINITY_DN1743_c1_g1), bZIP36 (TRINITY_DN4112_c0_g1), and bHLH4 (TRINITY_DN1499_c0_g1) were lower under blue light than in the control W. The expression of the 10 genes under different light qualities in *L. discolor* according to qRT-PCR was consistent with the transcriptome sequencing results.

## 4. Discussion

### 4.1. Light Quality Orchestrates the Biosynthesis of Phytohormones and Flavonoids in L. discolor

Light quality is a crucial environmental factor that not only drives photosynthesis but also serves as a primary signal regulating photomorphogenesis and metabolic activities [43]. In this study, most of the differential phytohormones of *L. discolor* were up-regulated under red light, resulting in the highest total relative content. In this study, most differentially accumulated phytohormones in *L. discolor* were up-regulated under red light, yielding the highest total relative content. This suggests that red light favors the overall accumulation of phytohormones, particularly gibberellins (GAs), jasmonic acid (JA), and auxins. Yellow and white light treatments followed in efficacy; specifically, yellow light maximized IAA and ACC contents, while white light induced the highest levels of castasterone, melatonin, and abscisic acid (ABA). Aligning with our findings, Wang [44] and OuYang [45] reported that red light promoted GA synthesis in *Panax ginseng* and *Picea abies*, respectively, whereas blue light decreased GA levels. These consistent trends indicate that light-quality-induced differences in phytohormone profiles stem from the specific light sensitivities of distinct hormone synthesis pathways.

Flavonoid biosynthesis is co-regulated by external light environments and intrinsic plant factors. In this study, 80 flavonoid metabolites were found to be significantly different in response to different light quality treatments, with the most metabolites significantly up-regulated under blue light, followed by green and red light; treatment with yellow light triggered the fewest up-regulated flavonoid metabolites. In the study by Chen [46], green, yellow and red light treatments were favorable for the accumulation of flavonoids, while blue light was unfavorable for the accumulation of flavonoids. These results are contradictory to the results of the present study, which may be the result of differences in the species used. In the present study, yellow light treatment was unfavorable for the accumulation of flavonoids but red and green light were advantageous for the accumulation of some specific flavonoid metabolites (Cyanidin 3-O-beta-D-sambubioside, etc.); however, it has also been noted that yellow light had no significant effect on the growth and synthesis of flavonoid compounds in *Tetrastigmatis hemsleyani* [47]. Furthermore, it has been found that blue light promotes the synthesis of flavonoid compounds [48,49] and that red light inhibits their synthesis [50]. Similarly, *L. discolor* significantly up-regulated the most differential flavonoids under blue light, mainly flavonoids, flavonols, and anthocyanins (Quercetin 3-O-[beta-D-xylosyl-(1->2)-beta-D-glucoside], Pelargonidin 3-O-(6-caffeoyl-beta-D-glucoside), etc.). Our results align with broader evidence demonstrating that blue light promotes the accumulation of these flavonoids. The results showed that flavonoid compounds in the leaves of *L. discolor* were affected by the light quality in a highly variable manner, further indicating the specificity of the response of different flavonoid metabolites to light quality, which provides a theoretical reference for the subsequent precision indoor cultivation of *L. discolor*. Building upon these insights, our specific LED-based cultivation parameters offer a reproducible ex situ conservation protocol for this endangered medicinal species, directly addressing wild population decline while maximizing its pharmacological value

### 4.2. Phytohormone-Mediated Modulation of Key Structural Genes in Flavonoid Biosynthesis

In this study, the expression levels of *L. discolor* leaves under different light quality treatments were mainly due to the expression of enzyme genes such as *4CL*, *PAL*, *CYP73A*, *FLS*, *CCoAOMT*, and *C12RT1*, which indicated that the synthesis of flavonoid compounds in *L. discolor* leaves was not under the control of a single gene but co-regulated by multiple genes. Among them, the two *PAL* enzyme genes were highly expressed under blue light and lowly expressed under green light. There was a positive correlation between *PAL* and Hesperetin, Dihydrokaempferol, Cyanidin 5-O-beta-D-glucoside 3-O-beta-D-sambubioside, and the metabolites. The relative contents of these metabolites showed similar trends under different light qualities, indicating that *L. discolor* can adapt to the light environment by enhancing *PAL* gene expression and *PAL* activity to promote downstream flavonoid metabolism under light stress. This is similar to the findings of Guo [51] and Zhang [52]. *4CL* was shown to be a key enzyme in flavonoid metabolism for the first time by Lin [53] in his investigation into the roles of the *Arabidopsis 4CL* gene family in flavonoid metabolism; additionally, *4CL3* was shown to be the key enzyme in flavonoid metabolism. In this study, the *4CL* in *L. discolor* leaves was highly expressed under green light and, in correlation analysis, there was a positive correlation between *4CL* and Hesperetin 7-O-glucoside. Therefore, it was hypothesized that the enhancement of *4CL* activity in *L. discolor* leaves under green light would in turn promote the production of Hesperetin 7-O-glucoside, thereby affecting the total flavonoid metabolic content. *CYP73A* expression in this study was down-regulated in blue light, green light, and yellow light. Correlation analysis showed that *CYP73A* was positively correlated with Phlorizin, Pelargonidin 5-O-beta-D-glucoside 3-O-beta-D-sambubioside, and Dihydrokaempferol and negatively correlated with Liquiritigenin, Prunin, Pelargonidin 3-O-glucoside, etc. We hypothesize that *L. discolor* leaves under blue light, green light, and yellow light lead to increasing levels of Liquiritigenin, Prunin, etc., by attenuating the expression of *CYP73A*, Pelargonidin 3-O-glucoside, and other metabolites.

*F3′H* is an important upstream gene in the flavonoid pathway. In the present study, the expression levels of *F3′H* were significantly up-regulated under blue, red, and yellow light treatments compared to white light, but *F3′5′H* expression was significantly down-regulated in blue light; this may be due to the different sensitivities of these two enzymes to light quality. Liao [54] showed, via in vitro functional analysis, that *CsCCoAOMT1* efficiently methylated the 6-, 7-, 8-, and 3′-hydroxyl groups of a variety of flavonoids with an o-hydroxyl group, with a strong preference for flavonols and flavonoids, and demonstrated, via cis-overexpression and viral-induced gene-silencing assays, that *CsCCoAOMT1* is a highly efficient multifunctional O-methyltransferase involved in the biosynthesis of polymethoxyflavones (PMFs) in *Citrus Sinensis*. Song [55] showed that overexpression of *CCoAOMT* contributes to lignin deposition and drought tolerance in *Pennisetum purpureum* by promoting the accumulation of flavonoid compounds in transgenic *Nicotiana tabacum*. In this study, *CCoAOMT* was significantly up-regulated in blue light and there was a significant positive correlation between *CCoAOMT* and the metabolites of Hesperetin, Quercetin 3-O-(6-O-malonyl-beta-D-glucoside), Glycitin, and (-)-Vestitol that were significantly up-regulated in blue light, indicating that the *CCoAOMT* enzyme gene of *L. discolor* positively regulates the synthesis of flavonoid metabolites under blue light and further confirming the involvement of *CCoAOMT* in flavonoid biosynthesis [56,57]. Some relevant findings suggest that overexpression of flavonoid-biosynthesis-related genes enhances flavonoid-mediated antioxidant activity to improve plant stress tolerance [58]. Rhamnosyltransferases are capable of transferring active rhamnosyl groups from nucleotide sugars to specific receptors involved in the production of secondary metabolites and play an irreplaceable role in the regulation of hormone levels, maintenance of cellular structure, signaling, and chemical defense in plants [59]. In this study, there were differences in the expression of C12RT1 under different light treatments, with C12RT1 being up-regulated in response to blue treatment. C12RT1 was significantly positively correlated with Peonidin 3-O-glucoside, Pelargonidin 3-O-(6-caffeoyl-beta-D-glucoside), Neohesperidin, and Cyanidin 5-O-beta-D-glucoside 3-O-beta-D-sambubioside, suggesting that, in *L. discolor,* C12RT1 promotes the accumulation of these substances under blue light. Combining the previous studies and preliminary speculation of this experiment, the 12 metabolic enzymes mined by differential expression analysis may play important roles in the physiological activities of *L. discolor* as a result of its response to different light quality stresses, i.e., increasing its activity and accelerating metabolic processes, thus promoting the biosynthesis and accumulation of flavonoids; this can improve the growth and development of *L. discolor*.

Multiple developmental processes in plants are induced by light signals and are affected by phytohormones. Several studies have shown that externally applied phytohormones or phytohormone analogs can affect the synthesis and accumulation of flavonoids [60,61]. The results of this study show that flavonoid metabolites and phytohormones are closely related in *L. discolor*. Reid [62] and Yamaguchi [63] suggested that red light could regulate the sensitivity of embryonic axial cells to GA by mediating the synthesis of GA through photosensitizing pigments (PHYs). In the results of this study, GA53, GA8, and DHZ were significantly up-regulated by red and yellow light, and the total relative content of phytohormones was also highest under red light. It was speculated that the accumulation of differential phytohormone metabolites in *L. discolor* under red light conditions, especially in the gibberellins, was similar to that found by Lei [64]. IAA is a natural growth hormone commonly found in plants, and its effect on the physiology of plant growth is dualistic, characterized by “promotion at low concentration and inhibition at high concentration” [65]. In addition, IAA was significantly down-regulated under green light, red light, and blue light, which was similar to the study by Huang [66]. This indicated that both red light and yellow light could significantly increase the content of GA and growth-hormone-like hormones. However, *L. discolor* showed significant down-regulation of IAA phytohormones under blue light, with the lowest total relative content, in which there was a significant negative correlation between IAA and Neohesperidin and Rotenone, suggesting that IAA may have an inhibitory effect on Neohesperidin and Rotenone or that the accumulation of these metabolites may hinder the increase in IAA. Previous studies have reported that the accumulation of flavonoids can hinder the polar transport of auxin [67,68], indicating that flavonoids play an important role in the regulation of auxin transport. However, the flavonoid substances involved in this process in plants need further exploration due to the complex nature of the terminal modification of these compounds. It has been shown that the regulatory effect of GA on flavonoid compounds is not only related to the type of gibberellin compound but also closely related to the plant species and gibberellin concentration [69,70]. Most of the gibberellin phytohormones and flavonoid compounds in this study exhibited significant positive correlations. GA24 was associated with Kaempferol 3-O-beta-D-glucosyl-(1->2)-beta-D-glucoside, Cyanidin 5-O-beta-D-glucoside 3-O-beta-D-sambubioside, and other flavonoids and flavonols and showed significant positive correlations anthocyanins, indicating that GA24 has a promoting effect on flavonoids and flavonols and anthocyanin metabolites, which is similar to the findings of Weng [71]. However, there was a negative correlation between GA36 and 2-Hydroxy-2,3-dihydrogenistein, a negative correlation between GA5 and Neohesperidin, and negative correlations between GA20 and quercetin 3-sophoridin and Pelargonidin 3-O-(6-caffeoyl-beta-D-glucoside). This may be due to the fact that gibberellins have different double bonds, hydroxyl numbers, and positions, which in turn affect the regulation of flavonoids in different ways and directions; it is hypothesized that these gibberellins negatively regulate the synthesis of these flavonoid metabolites [72].

As an effective scavenger of hydroxyl radicals in plant systems, MT plays an important role in enhancing plant resistance and improving plant growth and development [73,74]. In this study, MT showed highly significant positive correlations with various flavonoid metabolites such as (+)-Gallocatechin and Xanthohumol, similar to the findings of Song [75]. Song’s study found that melatonin treatment significantly induced the enrichment of flavonoid metabolites, mainly xanthohumol and its downstream metabolites, suggesting that melatonin plays an important role in promoting the biosynthesis of flavonoid compounds that have a positive effect on antioxidant and antistress effects in plants. The study by Yu [58] showed that an increased abundance of epigallocatechin may enhance the resistance of ginkgo (*Ginkgo biloba*) to water stress by reducing ROS accumulation; therefore, this study can further proves that MT positively regulates flavonoid metabolites and enhances plant stress resistance. Through correlation analysis, it was found that differential phytohormones regulate flavonoid-related differential metabolism in a multidirectional manner, and it is hypothesized that there may be a complex intrinsic regulatory relationship between these phytohormones and flavonoid metabolites, which further affects the accumulation of flavonoid metabolites.

### 4.3. Transcription Factors Act as Crucial Signal Transducers in the Phytohormone-Flavonoid Networ

Transcription factors (TFs) govern plant defense systems by binding to specific regulatory regions of target genes [76]. In this study, the regulatory prediction analysis of the predicted transcription factors and differential enzyme genes in the flavonoid biosynthesis pathway in different light-treated *L. discolor* leaves showed that bZIP53, bZIP2, bHLH4, and bHLH44 are the key transcription factors involved in the biosynthesis of flavonoids. R2R3-MYBs have been shown to regulate anthocyanin synthesis in ornamental plants by affecting the expression of structural genes. For example, AbMYB5 in R2R3-MYBs may be involved in the regulation of anthocyanin synthesis and metabolism in *Ananas comosus* leaves by promoting anthocyanin branching through up-regulating the expression of *CHS*, *F3H*, *F3′H*, and *ANS* [77]. In the present study, there was a significant positive correlation between MYB93 and *PAL* and *CYP73A*; therefore, it is hypothesized that MYB93 may affect the expression of flavonoids in *L. discolor* leaves by positively regulating *PAL* and *CYP73A*. This may be caused by differences in the regulatory regions of MYB as well as the differences in the roles of MYB in different species. bHLH plays an important regulatory role in flavonoid metabolism [78]; bHLH13, bHLH4, and bHLH16 are all members of the bHLH family. The bHLHs that have been reported to regulate flavonoid biosynthesis mainly achieve this through the regulation of flavonoid biosynthesis structural gene expression. bHLH16 positively regulated the expression of *4CL* and *FLS* in this study, which is similar to studies involving *Erigeron breviscapus* (EbbHLH80) [79], *Artemisia annua* (AabZIP19) [80], and *Myrica rubra* (MrbHLH1) [81]. The results were confirmed using functional and biochemical experiments to regulate the biosynthesis of flavonoid compounds via the direct regulation of the expression of structural genes for flavonoid biosynthesis. However, certain bHLH transcription factors inhibit flavonoid synthesis, probably because this IF competes with other TFs for the same cis-regulatory element or transcriptional repressor domain for its primary role. The bHLH4 in this study negatively regulated *PAL*, *C12RT1*, and *IF7MAT* enzyme genes to affecting the biosynthesis of flavonoid metabolites, which is similar to the findings reported by Wang [82], who found that MhbHLH130 directly binds to and represses the expression of MhCHS promoter and that repression of bHLH130 promotes the accumulation of flavonoids.

Transcription factors of the bZIP family are ubiquitous across plant species, serving crucial functions in secondary metabolism, photomorphogenesis, signal transduction, defense against pathogens, and overall growth and development. Our current research demonstrates that the enzyme genes *C12RT1* and *CCoAOMT* are positively regulated by bZIP44. Conversely, bZIP36 exerts a negative regulatory effect on *C12RT1*, *CYP81E*, and *IF7MAT*, whereas bZIP2 upregulates *CYP81E*. This aligns with previous findings by Wang [83], who reported that in transgenic tomato fruits and pear calli (using RNAi or E8 promoter-driven transient overexpression), PpbZIP44 directly targets the *PpADT* and *PpF3H* promoters. This interaction redirects carbon flux toward phenylalanine metabolism, thereby elevating flavonoid and phenylalanine levels. Similarly, during the flowering stage, UV radiation induces high expression of VvibZIPC22, which governs flavonol biosynthesis and promotes the accumulation of flavonol compounds and flavonol synthase [84]. Furthermore, FtbZIP genes have been shown to control flavonoid production by modulating F3H, a crucial enzyme gene in flavonoid synthesis [85]. Consistent with these previous reports, our results indicate that bZIP44, bZIP36, and bZIP2 transcriptionally control flavonoid biosynthesis by targeting the expression of key enzyme genes, specifically *C12RT1*, *CCoAOMT*, *CYP81E*, and *IF7MAT*. Ultimately, under varying light quality treatments, multiple transcription factors—including bZIP2, bZIP36, bZIP44, bZIP53, MYB88, MYB93, MYBCD5, bHLH4, and bHLH16—are likely implicated in the metabolic synthesis of flavonoids in *L. discolor* leaves.

The correlation between phytohormones and TFs was analyzed and the regulatory relationship of “phytohormone–transcription factor–metabolizing enzyme genes” was initially established, which further facilitated the analysis of the molecular mechanism of the phytohormone biosynthesis of flavonoids. In *Arabidopsis thaliana*, GAs negatively regulate anthocyanin accumulation and inhibit flavonol biosynthesis induced by low temperature or sucrose [62]. In this study, the expression of GA5, GA7, and GA20 was down-regulated under blue light; correlation analyses showed significant negative correlations between GA5 and bZIP44, GA7 and BZIP36, and GA20 and bZIP44/bZIP36, suggesting that blue light leads to a decrease in endogenous gibberellin concentration in *L. discolor* leaves, regulating bZIP44 and bZIP36 and, in turn, inducing the expression of *PAL*, *C12RT1*, *CCoAOMT*, and *IF7MAT*, which led to the accumulation of flavonoids. This was similar to the findings in the study by Song [86], where *Vaccinium corymbosum* was subjected to UV-B radiation, resulting in a decrease in endogenous gibberellin concentration that affected the accumulation of flavonoids via affecting the expression of MYB20, MYB44, and VcMYB14. However, in this study, GA24 was significantly associated with MYB 93 and bHLH 4; the use of different species could be a reason for any differences in the mechanisms by which transcription factors exert their effects. In addition, UV-B radiation may reduce the endogenous growth hormone concentration and decrease the expression levels of ARF18 and IAA, resulting in increased expression of MYB114 or decreased expression of MYB20 and MYB44. In this study, the expression of the growth phytohormones IAA, ABA, and BR was down-regulated under blue light treatment; correlation analyses showed significant negative correlations between IAA, ABA, and bZIP44; BR was significantly negatively correlated with bZIP44 and bZIP2. Therefore, blue light decreases the content of the phytohormones IAA, ABA, and BR to regulate TFs in *L. discolor*, resulting in the up-regulation of the expression of enzyme genes such as *PAL*, *C12RT1*, *CCoAOMT*, and *CYP81E*.

In this study, upon cis-acting response element analysis, a total of four GA-responsive elements (P-box/GARE-motif/TATC-box) were present in MYBCD5 and MYB88, and AUX-responsive elements were also present in MYB88 and MYB93. These results are similar to the findings of Sun [87]. The cis-acting elements predicted to be associated with the initiation of StMYB cis-acting elements in the region include the MeJA-responsive element, ABA-responsive element, gibberellic acid-responsive element, salicylic acid-responsive element, and Auxin-responsive element; additionally, the differences in the GA-responsive elements of MYBCD5 and MYB88 suggest that they may be involved in different hormone signaling pathways. Auxin response elements are involved in the regulation of many processes, such as tropism, apical dominance, and abiotic stress, via regulating the transcription of downstream target genes through the specific binding of growth hormone response elements [88]. In the study by Jiang [89], the growth hormone response factor ARF2 acted upstream of MYB12 to increase the flavonol content by regulating the expression of flavonol branching pathway genes, such as *F3′H* and *FLS*. On the other hand, ARF2 also acted upstream of *TT2*, a key gene for the regulation of proanthocyanidins, and interacted with the *TT2* protein to increase the flavonol content via the regulation of *ANS*. These regulatory mechanisms can enable plants to better adapt to external environmental changes and facilitate the accumulation of active ingredients. Therefore, it can be hypothesized that the IF promoter, which is related to flavonoid biosynthesis, is regulated by phytohormones (AUX, GA, ABA), it may be involved in regulating the expression of key enzyme genes of the flavonoid biosynthesis pathway and thus play a role in the regulatory network of “phytohormone–IF–enzyme gene expression”.

To validate these transcriptomic findings, qRT-PCR was performed on key TFs and structural genes. The expression trends across different light qualities were highly consistent with the sequencing data, confirming its reliability. This process enhanced the *L. discolor* flavonoid content, affecting plant growth and development. These experiments provide insights into the regulation of plant secondary metabolism accumulation in response to environmental light characteristics, as well as data on the molecular regulatory network of phytohormones involved in flavonoid compound synthesis. From an evolutionary perspective, comparative analysis with other Orchidaceae species reveals conserved light-responsive TF modules. However, the unique flavonoid profile and specific TF-target correlations observed in *L. discolor* highlight a fascinating lineage-specific metabolic diversification, likely driven by its adaptation to the low-light understory environments of its native habitat.

## 5. Conclusions

**Figure 14:**
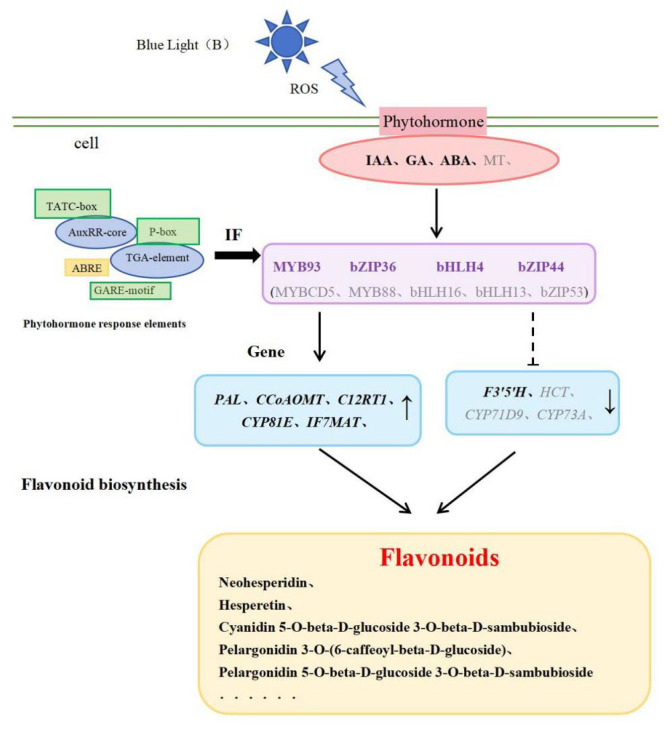
Model of regulatory relationship between phytohormones and flavonoid metabolites under blue light. Note: Arrows next to gene names represent increased (up-regulated) or decreased (down-regulated). Solid arrows and dotted blunt lines indicate activation and suppression, respectively. The inferred key phytohormones, transcription factors (TFs), enzyme genes (Genes), and flavonoid metabolites (Flavonoids) are indicated for *L. discolor*.

By integrating transcriptomic and metabolomic analyses, this study elucidated the light-quality-dependent regulatory networks governing phytohormone and flavonoid biosynthesis in *L. discolor*. We found that blue and red light generally promoted flavonoid accumulation, whereas yellow light was inhibitory. Furthermore, red light distinctively enhanced the accumulation of specific phytohormones—notably gibberellins, jasmonic acid, and auxins—while both red and green light effectively induced targeted flavonoid metabolites. Multi-omics co-expression analysis identified MYB93, bZIP36, bHLH4, and bZIP44 as pivotal regulators. These transcription factors function as key nodes in the “phytohormone–TF–structural gene” network, predicted to integrate AUX, GA, and ABA signals to modulate flavonoid biosynthesis. Based on these findings, we propose a regulatory model for the blue light response in *L. discolor* (Figure 14). Under blue light illumination, induced reactive oxygen species (ROS) trigger a signaling cascade that modulates the synthesis of IAA, GA, and ABA. This hormonal activation drives specific TFs to upregulate structural genes, ultimately promoting the biosynthesis of key flavonoids, including neohesperidin, hesperetin, and pelargonidin derivatives.

## Figures and Tables

**Table 1:** Primers for qRT-PCR.

Gene	Gene ID	Forward Primer (5′-3′)	Reverse Primer (5′-3′)
*26SrRNA*	-	CTGATTTCCAGTGCGAATACGA	TCCGAACGACTAAAGGATCGA
*PAL*	TRINITY_DN2373_c0_g2	GGCTGATAGGGCGAAGGTT	CGATGCCATTGCTGATACTGT
*C12RT1*	TRINITY_DN3551_c0_g1	GTCCCTGTTGGTGCTCTTG	TTCCTGGCGACCGTAAAA
*CCoAOMT*	TRINITY_DN9119_c0_g1	ATAACCGCCATCGACATCT	GAGCATCATCTCGCCGTAG
*CYP81E*	TRINITY_DN759_c1_g1	CTGTGCCTCACTACCCATTG	GAACCTCATCCGCCCTTAT
*IF7MAT*	TRINITY_DN43293_c0_g1	GCCACCACAAATGAAGAAATG	GGAGCGAGCAAAGAAGGAG
*F3′5′H*	TRINITY_DN16239_c0_g1	CGCCATTGAACGAACCAG	GCAAAGTATCCAGCCTCCAC
*bHLH4*	TRINITY_DN1499_c0_g1	AGGAAGTGACTGACACCGAATG	TACCGGGATGCAGACCAAG
*MYB93*	TRINITY_DN6460_c0_g1	ACACCCACCTGAAGAAGAAGC	GGAGGATGTATTGAAGGCACTG
*bZIP44*	TRINITY_DN1743_c1_g1	AAACCACCATCTTTCTACCGC	GGAAGGCAACATCAATCAGG
*bZIP36*	TRINITY_DN4112_c0_g1	CCTTCTAAGCCCTCTATCTACG	GCGATTCAATGCAAACTGG

## Data Availability

The data are contained within the article.

## Supplementary Materials

The following supporting information can be downloaded at: https://www.mdpi.com/article/10.3390/genes17040445/s1, Figure S1: Leaves of ‘Min Hot Round Shuai’; Table S1: Endogenous hormones in the leaves of *Ludisia discolor* under different light quality treatments; Table S2: Flavonoid metabolites in the leaves of *Ludisia discolor* under different light quality treatments; Table S3: Correlation of “Endogenous hormone-flavonoids metabolites” in the leaves of *Ludisia discolor*; Table S4: Sample sequencing data to evaluate tables; Table S5: Assembly result statistics; Table S6: Enzyme genes involved in flavonoid biosynthesis of in the leaves of *Ludisia discolor*; Table S7: Correlation analysis of differential flavonoid metabolites; Table S8: Transcription factor families with current 10 gene numbers; Table S9: Correlation of “Transcription factor-Flavonoid differentially expressed genes” in the leaves of *Ludisia discolor*; Table S10: Correlation of “Endogenous hormone-Transcription factor” in the leaves of *Ludisia discolor*; Table S11: Correlation of “Endogenous hormone-Transcription factor” in the leaves of *Ludisia discolor*; Table S12: Conditions of flavonoids-related transcription factors in the leaves of *Ludisia discolor*.
