# Supplementary material for: Multi-Omics Reveals Light-Quality-Dependent Phytohormone and Transcription Factor Networks Regulating Flavonoid Biosynthesis in Ludisia discolor
Source: Genes (Basel). 2026 Apr 13;17(4):445. doi: 10.3390/genes17040445 (PMC13115993; doi:10.3390/genes17040445)
Supplement: Supplementary file 1 [file genes-17-00445-s001.zip › genes-4247973-Supplementary Materials-Table.pdf]

TableS1 Endogenous hormones in the leaves of *Ludisia discolor* under different light quality treatments

NO	metabolite		W	B	R	G	Y
1	Indole-3-acetate	IAA	253.63±9.05	208.89±4.29	229.63±5.79	204.28±3.87	237.75±11.59
2	Gibberellin A24	GA24	46.99±4.97	297.78±18.68	80.11±12	71.35±13.44	48.51±1.54
3	Gibberellin A53	GA53	268.13±68.99	883.74±711.94	553.53±124.23	167.6±28.21	716.02±115.32
4	Gibberellin A4	GA4	331.23±2.46	300.56±54.53	223.12±19.72	244.35±8.51	217.19±8.58
5	Gibberellin A36	GA36	416.45±17.17	584±99.12	339.02±29.37	326.19±88.93	322.32±121.7
6	Gibberellin A5	GA5	101.41±11.01	59.34±4.98	83.53±8.73	61.88±0.72	76.79±5.15
7	Gibberellin A7	GA7	101.5±4.94	51.01±0.97	106.9±7.46	61.81±6.99	115.45±12.33
8	Gibberellin A8	GA8	745.22±35.6	806.77±9.39	1505.05±88.33	1176.77±18.71	1259.43±48.79
9	Gibberellin A20	GA20	569.25±74.15	284.42±14.45	645.24±54.42	397.83±34.26	581.06±123.9
10	1-Aminocyclopropane -1-carboxylate	ACC	455.21±22.57	250.33±18.76	359.52±40.51	654.07±27.63	348.76±34.41
11	Melatonin	MT	366.76±48.95	104.33±39.9	216.39±22.26	248.23±89.34	162.93±40.23
12	Dihydrozeatin riboside	DZR	77.2±4.57	61.94±6.07	65.04±8.7	81.57±9.85	79.65±6.63
13	Dihydrozeatin	DHZ	6734.95±221.51	2371.43±93.27	8577.13±288.61	5418.74±59.35	7667.98±424.05
14	trans-Zeatin	tZT	83.35±5.12	55.5±11.13	76.95±13.51	66.58±18.62	75.34±13.6
15	Zeatin	ZT	16593.55±261.59	12626.06±788.22	17279.46±694.39	14296.77±688.25	14852.33±456.09
16	cis-Zeatin riboside	cZR	10.05±3.59	30.37±16.73	4.36±2.05	6.72±1.31	1.4±1.24
17	Abscisic acid	ABA	223.16±7.42	180.72±2.33	202.62±7.14	215.81±16.55	212.46±6.52
18	Castasterone	BR	827.41±48.48	461.52±45.83	722.06±42.4	600.67±17.29	658.82±54.04

TableS2 Flavonoid metabolites in the leaves of *Ludisia discolor* under different light quality treatments

Class	NO	metabolite	W_vs_B	W_vs_R	W_vs_G	W_vs_Y	KEGG annotation
Flavanone	1	Pinostrobin	unchanged	unchanged	unchanged	up	C16419
	2	Hesperetin 7-O-glucoside	unchanged	up	unchanged	unchanged	C16422
	3	Neohesperidin	up	up	unchanged	up	C09806
	4	Prunin	unchanged	unchanged	up	down	C09099
	5	Liquiritigenin	unchanged	unchanged	unchanged	down	C09762
	6	Hesperetin	unchanged	unchanged	unchanged	up	C01709
	7	Naringenin	unchanged	up	up	down	C09099

		7-O-beta-D-glucoside					
	8	Naringin	unchanged	down	unchanged	down	C09789
	9	Sakuranetin	unchanged	up	unchanged	unchanged	C09833
	10	Dihydrokaempferol	down	unchanged	unchanged	unchanged	C00974
Flavanol	1	(-)-Epicatechin	down	unchanged	unchanged	down	C09727
	2	(+)-Gallocatechin	down	unchanged	down	down	C12127
Chalcone and Dihydrochalcone	1	Phlorizin chalcone	down	down	down	down	C16406
	2	2',3,4,4',6'-Peptahydroxychalcone 4'-O-glucoside	down	down	down	down	C16408
	3	Phlorizin	down	down	unchanged	down	C01604
	4	Desmethylxanthohumol	up	up	unchanged	up	C16416
	5	Xanthohumol	down	down	down	down	C16417
Flavone and Flavonol	1	Myricetin	down	unchanged	unchanged	down	C10107
	2	Kaempferol 3-O-beta-D-glucosyl-(1->2)-beta-D-glucoside	up	up	unchanged	up	C12634
	3	Apigenin 7-O-[beta-D-apiosyl-(1->2)-beta-D-glucoside]	up	unchanged	up	up	C04858
	4	Kaempferol 3-sophorotrioside	up	up	up	up	C12635
	5	Quercetin 3-O-[beta-D-xylosyl-(1->2)-beta-D-glucoside]	down	down	down	up	C12637
	6	Malonylapiin	unchanged	unchanged	unchanged	up	C05622
	7	Quercetin	unchanged	down	unchang	down	C05623

	3-O-glucoside			ed		
8	Kaempferol-3-O-rutinoside	down	unchanged	unchanged	unchanged	C21833
9	Quercetin 3-O-beta-D-glucosyl-(1->2)-beta-D-glucoside	down	unchanged	unchanged	down	C12667
10	3-O-Methylquercetin	down	down	down	down	C04443
11	Astragalin	down	down	unchanged	down	C12249
12	Scolymoside	down	up	unchanged	unchanged	C12630
13	Rutin	down	unchanged	down	down	C05625
14	Apiin	up	up	up	up	C04858
15	Luteolin 7-O-glucuronide	down	unchanged	unchanged	down	C03515
16	Luteolin 7-O-[beta-D-glucuronosyl-(1->2)-beta-D-glucuronide]	up	up	up	up	C12632
17	Isoswertisin 2''-rhamnoside	up	unchanged	up	up	C12629
18	Quercetin 3-O-(6-O-malonyl-beta-D-glucoside)	unchanged	unchanged	unchanged	up	C12638
19	Acacetin	down	unchanged	unchanged	unchanged	C01470
20	Luteolin 7-O-[beta-D-glucuronosyl-(1->2)-beta-D-glucuronide]-4'-O-beta-D-glucuronide	unchanged	unchanged	down	down	C04900
21	Quercetin 3-(2G-xylosylrutinoside)	up	up	unchanged	unchanged	C10175
22	Kaempferol 3-O-[6-(4-coumaroyl)-beta-D-glucosyl-(1->2)-beta-D-glucosyl-(1->2)-beta-D-glucoside]	down	unchanged	down	down	C12636
23	Vitexin 2''-O-beta-L-rhamnoside	down	down	down	down	C12628
24	Quercetin	unchanged	unchang	unchang	up	C12668

		3-O-beta-D-glucosyl-(1->2)-beta-D-glucosyl-(1->2)-beta-D-glucoside		ed	ed		
	25	Luteolin 7-O-beta-D-glucoside	down	down	unchanged	down	C03951
	26	Syringetin	down	unchanged	unchanged	unchanged	C11620
Isoflavone	1	Glyceocarpin	up	up	unchanged	unchanged	C01701
	2	(-)-Glyceollin I	up	unchanged	unchanged	up	C16224
	3	Medicarpin 3-O-glucoside-6'-malonate	down	unchanged	unchanged	down	C16222
	4	Formononetin 7-O-glucoside-6''-O-malonate	down	unchanged	unchanged	down	C16195
	5	Glycitin	unchanged	unchanged	up	up	C16225
	6	(-)-Vestitol	unchanged	unchanged	unchanged	up	C07593
	7	Rotenone	unchanged	up	unchanged	unchanged	C16197
	8	Malonylglycitin	down	down	down	down	C12631
	9	2-Hydroxy-2,3-dihydrogenistein	unchanged	up	up	down	C19727
	10	5-Hydroxypseudobaptigenin	unchanged	unchanged	unchanged	up	C05376
	11	Biochanin A-beta-D-glucoside	down	down	unchanged	down	C12625
	12	Biochanin A 7-O-(6-O-malonyl-beta-D-glucoside)	down	down	down	down	C05376
	13	Sissotrin	down	down	up	up	C16188
	14	7-Hydroxy-2',4',5'-trimethoxyisoflavone	down	down	down	down	C16191
	15	Malonyldaidzin	down	down	down	down	C10503
	16	Medicarpin	down	unchanged	down	down	C10216
	17	Daidzin	down	down	down	down	C00858
	18	Formononetin	down	unchanged	down	down	C10509
	19	Ononin	unchanged	down	down	down	C16223
	20	(-)-Medicocarpin	unchanged	up	unchang	unchang	

					ed	ed	
Anthocyanidin	1	Pelargonidin	down	down	down	down	C05904
	2	Cyanidin 3-glucoside 5-caffeoylglucoside	down	down	down	down	C16372
	3	Cyanidin 5-O-beta-D-glucoside 3-O-beta-D-sambubioside	down	unchanged	unchanged	up	C20493
	4	Cyanidin 3-O-beta-D-sambubioside	unchanged	up	up	unchanged	C20490
	5	Delphinidin 5-O-beta-D-glucoside 3-O-beta-D-sambubioside	unchanged	down	up	down	C20494
	6	Pelargonidin 3-O-beta-D-sambubioside	unchanged	unchanged	unchanged	up	C20489
	7	Pelargonidin 3-O-rutinoside 5-O-beta-D-glucoside	up	up	unchanged	up	C12645
	8	Malvidin-3-(p-coumaroyl)-rutinoside-5-glucoside	down	down	down	down	C16296
	9	Cyanidin 3-O-rutinoside 5-O-beta-D-glucoside	down	down	unchanged	down	C12646
	10	Peonidin-3-(p-coumaroyl)-rutinoside-5-glucoside	down	down	down	down	C16293
	11	Peonidin 3-O-glucoside	unchanged	unchanged	up	up	C12141
	12	Pelargonidin 3-O-(6-caffeoyl-beta-D-glucoside)	unchanged	up	unchanged	up	C16297
	13	Cyanidin 3-O-(6-O-p-coumaroyl) glucoside	unchanged	down	down	unchanged	C12095
	14	Pelargonidin 5-O-beta-D-glucoside 3-O-beta-D-sambubioside	unchanged	unchanged	down	up	C20492

15	Petunidin 3-O-glucoside	down	down	down	down	C12139
16	Cyanidin 3-O-(2-O-beta-D-glucuronosyl)-beta-D-glucoside	unchanged	up	unchanged	down	C19762
17	Pelargonidin 3-O-glucoside	unchanged	up	unchanged	down	C12137

TableS3 Correlation of "Endogenous hormone - flavonoids metabolites" in the leaves of *Ludisia discolor*

No	Hormone		KO	Correlation
1	IAA	Neohesperidin	C09806	-0.917*
2	IAA	Phlorizin chalcone	C16406	0.983**
3	IAA	Kaempferol 3-sophorotrioside	C12635	-0.939*
4	IAA	Rotenone	C07593	-0.958*
5	IAA	Cyanidin 3-O-beta-D-sambubioside	C20490	-0.972**
6	GA24	Liquiritigenin	C09762	-0.964**
7	GA24	Naringin	C09789	-0.913*
8	GA24	Kaempferol 3-O-beta-D-glucosyl-(1->2)-beta-D-glucoside	C12634	0.99**
9	GA24	Apigenin 7-O-[beta-D-apiosyl-(1->2)-beta-D-glucoside]	C04858	0.967**
10	GA24	Quercetin 3-O-beta-D-glucosyl-(1->2)-beta-D-glucoside	C12667	0.959**
11	GA24	Isoswertisin 2''-rhamnoside	C12629	0.907*
12	GA24	Glycitin	C16195	0.992**
13	GA24	(-)-Vestitol	C16225	0.982**
14	GA24	5-Hydroxypseudobaptigenin	C19727	0.96**
15	GA24	Cyanidin 5-O-beta-D-glucoside 3-O-beta-D-sambubioside	C20493	0.962**
16	GA24	Malvidin-3-(p-coumaroyl)-rutinoside-5-glucoside	C16296	-0.956*
17	GA24	Pelargonidin 3-O-(6-caffeoyl-beta-D-glucoside)	C16297	0.94*
18	GA53	Hesperetin	C01709	0.924*

19	GA53	Quercetin 3-O-beta-D-glucosyl-(1->2)-beta-D-glucoside	C12667	-0.905*
20	GA53	Rutin	C05625	-0.929*
21	GA53	Malonyldaidzin	C16191	-0.944*
22	GA4	Luteolin 7-O-[beta-D-glucuronosyl-(1->2)-beta-D-glucuronide]	C12632	-0.969**
23	GA36	Prunin	C09099	-0.963**
24	GA36	Liquiritigenin	C09762	-0.918*
25	GA36	Dihydrokaempferol	C00974	0.963**
26	GA36	Quercetin 3-O-beta-D-glucosyl-(1->2)-beta-D-glucoside	C12667	0.967**
27	GA36	Quercetin 3-O-(6-O-malonyl-beta-D-glucoside)	C12638	0.955*
28	GA36	Acacetin	C01470	0.922*
29	GA36	(-)-Vestitol	C16225	0.93*
30	GA36	2-Hydroxy-2,3-dihydrogenistein	C12631	-0.901*
31	GA36	5-Hydroxypseudobaptigenin	C19727	0.974**
32	GA36	Cyanidin 5-O-beta-D-glucoside 3-O-beta-D-sambubioside	C20493	0.972**
33	GA36	Pelargonidin 3-O-glucoside	C12137	-0.922*
34	GA5	Neohesperidin	C09806	-0.933*
35	GA5	Phlorizin chalcone	C16406	0.948*
36	GA5	Phlorizin	C01604	0.959*
37	GA5	Kaempferol 3-sophorotrioside	C12635	-0.944*
38	GA5	3-O-Methylquercetin	C04443	0.924*
39	GA5	Vitexin 2''-O-beta-L-rhamnoside	C12628	0.966**
40	GA5	Rotenone	C07593	-0.989**
41	GA5	Biochanin A-beta-D-glucoside	C05376	0.95*
42	GA5	Cyanidin 3-O-beta-D-sambubioside	C20490	-0.984**
43	GA7	Naringin	C09789	0.936*
44	GA7	Ononin	C10509	0.907*
45	GA7	Pelargonidin 3-O-(6-caffeoyl-beta-D-glucoside)	C16297	-0.903*
46	GA20	Naringin	C09789	0.934*
47	GA20	Quercetin 3-O-glucoside	C05623	0.944*
48	GA20	Quercetin 3-O-[beta-D-xylosyl-(1->2)-beta-D-glucoside]	C12637	-0.93*
49	GA20	Ononin	C10509	0.914*

50	GA20	Pelargonidin 3-O-beta-D-sambubioside	C20489	-0.954*
51	GA20	Cyanidin 3-O-rutinoside 5-O-beta-D-glucoside	C12646	0.909*
52	GA20	Pelargonidin 3-O-(6-caffeoyl-beta-D-glucoside)	C16297	-0.936*
53	MT	(-)-Epicatechin	C09727	0.903*
54	MT	(+)-Gallocatechin	C12127	0.955*
55	MT	2',3,4,4',6'-Peptahydroxychalcone 4'-O-glucoside	C16408	0.933*
56	MT	Xanthohumol	C16417	0.996**
57	MT	Myricetin	C10107	0.93*
58	MT	3-O-Methylquercetin	C04443	0.933*
59	MT	Kaempferol 3-O-[6-(4-coumaroyl)-beta-D-gluc osyl-(1->2)-beta-D-glucosyl-(1->2)-beta-D-glucoside]	C12636	0.917*
60	MT	Medicarpin 3-O-glucoside-6'-malonate	C16224	0.956*
61	MT	Formononetin 7-O-glucoside-6''-O-malonate	C16222	0.941*
62	MT	Biochanin A 7-O-(6-O-malonyl-beta-D-glucosid e)	C12625	0.977**
63	MT	7-Hydroxy-2',4',5'-trimethoxyisofl avone	C16188	0.995**
64	MT	Malonyldaidzin	C16191	0.975**
65	MT	Medicarpin	C10503	0.915*
66	MT	Daidzin	C10216	0.935*
67	DZR	Luteolin 7-O-[beta-D-glucuronosyl-(1->2)-b eta-D-glucuronide]	C12632	0.946*
68	DZR	Sissotrin	C05376	-0.95*
69	DHZ	Prunin	C09099	0.911*
70	DHZ	Naringin	C09789	0.96**
71	DHZ	Quercetin 3-O-glucoside	C05623	0.912*
72	DHZ	Quercetin 3-O-[beta-D-xylosyl-(1->2)-beta-D-glucoside]	C12637	-0.93*
73	DHZ	Cyanidin 5-O-beta-D-glucoside 3-O-beta-D-sambubioside	C20493	-0.906*
74	DHZ	Pelargonidin 3-O-beta-D-sambubioside	C20489	-0.91*
75	DHZ	Pelargonidin	C16297	-0.938*

		3-O-(6-caffeoyl-beta-D-glucoside)		
76	tZT	Neohesperidin	C09806	-0.972**
77	tZT	Malonylapiin	C05622	-0.928*
78	tZT	Quercetin 3-O-glucoside	C05623	0.976**
79	tZT	Ononin	C10509	0.992**
80	tZT	Cyanidin 3-O-beta-D-sambubioside	C20490	-0.934*
81	tZT	Malvidin-3-(p-coumaroyl)-rutinoside-5-glucoside	C16296	0.921*
82	tZT	Cyanidin 3-O-rutinoside 5-O-beta-D-glucoside	C12646	0.904*
83	tZT	Pelargonidin 3-O-(6-caffeoyl-beta-D-glucoside)	C16297	-0.968**
84	ZT	Malonylapiin	C05622	-0.961**
85	ZT	Quercetin 3-O-glucoside	C05623	0.97**
86	ZT	Quercetin 3-O-[beta-D-xylosyl-(1->2)-beta-D-glucoside]	C12637	-0.982**
87	ZT	Pelargonidin 3-O-beta-D-sambubioside	C20489	-0.981**
88	ZT	Cyanidin 3-O-rutinoside 5-O-beta-D-glucoside	C12646	0.999**
89	cZR	Prunin	C09099	-0.956*
90	cZR	Liquiritigenin	C09762	-0.91*
91	cZR	Naringin	C09789	-0.934*
92	cZR	Dihydrokaempferol	C00974	0.933*
93	cZR	Kaempferol 3-O-beta-D-glucosyl-(1->2)-beta-D-glucoside	C12634	0.928*
94	cZR	Quercetin 3-O-beta-D-glucosyl-(1->2)-beta-D-glucoside	C12667	0.958*
95	cZR	Acacetin	C01470	0.956*
96	cZR	Glycitin	C16195	0.907*
97	cZR	(-)-Vestitol	C16225	0.936*
98	cZR	5-Hydroxypseudobaptigenin	C19727	0.97**
99	cZR	Cyanidin 5-O-beta-D-glucoside 3-O-beta-D-sambubioside	C20493	0.998**
100	ABA	Liquiritigenin	C09762	0.914*
101	ABA	Hesperetin	C01709	-0.953*
102	ABA	Myricetin	C10107	0.931*
103	ABA	Kaempferol 3-O-beta-D-glucosyl-(1->2)-beta-D-glucoside	C12634	-0.921*

104	ABA	Apigenin 7-O-[beta-D-apiosyl-(1->2)-beta-D-glucoside]	C04858	-0.917*
105	ABA	Isoswertisin 2''-rhamnoside	C12629	-0.991**
106	ABA	Luteolin 7-O-[beta-D-glucuronosyl-(1->2)-beta-D-glucuronide]	C12632	0.939*
107	ABA	Glycitin	C16195	-0.967**
108	ABA	(-)-Vestitol	C16225	-0.934*
109	ABA	Malvidin-3-(p-coumaroyl)-rutinoside-5-glucoside	C16296	0.967**
110	ABA	Peonidin-3-(p-coumaroyl)-rutinoside-5-glucoside	C16293	-0.903*
111	BR	Neohesperidin	C09806	-0.963**
112	BR	Malonylapiin	C05622	-0.934*
113	BR	Quercetin 3-O-glucoside	C05623	0.966**
114	BR	3-O-Methylquercetin	C04443	0.939*
115	BR	Vitexin 2''-O-beta-L-rhamnoside	C12628	0.914*
116	BR	Formononetin 7-O-glucoside-6''-O-malonate	C16222	0.915*
117	BR	Biochanin A 7-O-(6-O-malonyl-beta-D-glucoside)	C12625	0.913*
118	BR	Ononin	C10509	0.954*
119	BR	Cyanidin 3-O-beta-D-sambubioside	C20490	-0.941*
120	BR	Malvidin-3-(p-coumaroyl)-rutinoside-5-glucoside	C16296	0.915*
121	BR	Cyanidin 3-O-rutinoside 5-O-beta-D-glucoside	C12646	0.919*
122	BR	Pelargonidin 3-O-(6-caffeoyl-beta-D-glucoside)	C16297	-0.915*

TableS4 Sample sequencing data to evaluate tables

Sample	Reads Number	Base Number	GC Content(%)	Q30(%)
W-1	23,625,299	7,069,843,848	46.98%	93.04%
W-2	24,926,790	7,460,602,212	47.12%	93.83%
W-3	21,376,963	6,394,713,724	47.24%	93.89%
R-1	19,594,779	5,866,005,162	47.42%	92.95%
R-2	25,083,122	7,507,066,632	47.32%	93.01%
R-3	23,295,602	6,969,754,308	47.39%	93.97%
B-1	21,803,560	6,527,284,728	47.69%	92.41%
B-2	20,423,962	6,113,140,060	47.63%	93.35%
B-3	22,925,693	6,859,872,076	47.62%	92.80%

Y-1	19,979,355	5,979,738,688	47.32%	93.15%
Y-2	20,415,279	6,111,157,474	46.95%	93.47%
Y-3	20,168,863	6,036,051,238	47.39%	93.64%
G-1	21,134,377	6,325,698,672	46.50%	93.39%
G-2	19,409,338	5,808,513,680	47.08%	93.88%
G-3	19,589,246	5,861,189,574	47.08%	93.67%

Note: Read Number, pair-end Reads number in Clean Data;Base Number, total base number in Clean Data;GC Content,the percentages of G and C in Clean Data constitute the total base; Q30,the percentage of bases that Clean Data quality is greater than or equal to 30.

TableS5 Assembly result statistics

Length Range	Transcript	Unigene
200-300	22,711(15.53%)	18,663(26.70%)
300-500	21,442(14.66%)	14,520(20.77%)
500-1000	26,997(18.46%)	13,727(19.64%)
1000-2000	33,304(22.77%)	10,528(15.06%)
2000+	41,795(28.58%)	12,459(17.82%)
Total Number	146,249	69,897
Total Length	235,168,317	81,729,987
N50 Length	2,739	2,428
Mean Length	1608.00	1169.29

Note，Length Range,the different length intervals of Unigene, the number in the table represents the number of Unigenes in the corresponding interval, and the percentage in parentheses represents the number of Unigenes in the corresponding length interval.Total Number, the total number of Unigenes assembled; Total Length, the total length of Unigene assembled; N50 Length, the length of Unigene N50; Mean Length, the average length of Unigene.

TableS6 Enzyme genes involved in flavonoid biosynthesis of in the leaves of *Ludisia discolor*

Kegg pathway	No	Gene name	Enzyme number	KO	Gene function	Number
Ko00940 Phenylpropanoid biosynthesis	1	4-coumarate--CoA ligase	[EC:6.2.1.12]	K01904	4CL	12
	2	phenylalanine ammonia-lyase	[EC:4.3.1.24]	K10775	PAL	4
	3	trans-cinnamate 4-monooxygenase	[EC:1.14.14.91]	K00487	CYP73A	4
Ko00941 Flavonoid biosynthesis	1	shikimate O-hydroxycinnamoyltransferase	[EC:2.3.1.133]	K13065	HCT	12
	2	chalcone synthase	[EC:2.3.1.74]	K00660	CHS	10
	3	chalcone isomerase	[EC:5.5.1.6]	K01859	CHI	8

	4	caffeoyl-CoA O-methyltransferase	[EC:2.1.1.104]	K00588	CCOAOMT	7
	5	trans-cinnamate 4-monooxygenase	[EC:1.14.14.91]	K00487	CYP73A	4
	6	flavanone 7-O-glucoside 2''-O-beta-L-rhamnosyltransferase	[EC:2.4.1.236]	K13080	C12RT1	4
	7	phlorizin synthase	[EC:2.4.1.357]	K22845	PGT1	3
	8	flavonoid 3'-monooxygenase	[EC:1.14.14.82]	K05280	CYP75B1	3
	9	flavonol synthase	[EC:1.14.20.6]	K05278	FLS	2
	10	anthocyanidin reductase	[EC:1.3.1.77]	K08695	ANR	1
	11	flavonoid 3',5'-hydroxylase	[EC:1.14.14.81]	K13083	CYP75A	1
	12	5-O-(4-coumaroyl)-D-quinate 3'-monooxygenase	[EC:1.14.14.96]	K09754	CYP98A	1
	13	naringenin 3-dioxygenase	[EC:1.14.11.9]	K00475	F3H	1
Ko00942 Anthocyanin biosynthesis	1	flavonoid 6-hydroxylase	[EC:1.14.13.-]	K21383	3AT	1
ko00943 Isoflavonoid biosynthesis	1	isoflavone/4'-methoxyisoflavone 2'-hydroxylase	[EC:1.14.14.90 1.14.14.89]	K13260	CYP81E	6
	2	isoflavone 7-O-glucoside-6''-O-malonyltransferase	[EC:2.3.1.115]	K13264	IF7MAT	4
	3	flavonoid 6-hydroxylase	[EC:1.14.13.-]	K13267	CYP71D9	1
ko00944 Flavone and flavonol biosynthesis	1	flavonoid 3',5'-hydroxylase	[EC:1.14.14.81]	K13080	C12RT1	4
	2	isoflavone 7-O-glucoside-6''-O-malonyltransferase	[EC:2.3.1.115]	K13264	IF7MAT	4
	3	flavonoid 3'-monooxygenase	[EC:1.14.14.82]	K05280	CYP75B1	3
	4	flavonol-3-O-glucoside L-rhamnosyltransferase	[EC:2.4.1.159]	K22772	FG2	1
	5	flavonoid 3',5'-hydroxylase	[EC:1.14.14.81]	K13083	CYP75A	1

6	flavonol-3-O-L-rhamnoside -7-O-glucosyltransferase	[EC:2.4.1.-]	K2277 1	UGT73C 6	1

Table S7 Correlation analysis of differential flavonoid metabolites

No	Enzyme gene		Flavonoid metabolites		Correlation
1	TRINITY_DN2373_c0_g4	PAL-2	Liquiritigenin	C09762	-0.948*
2	TRINITY_DN2373_c0_g4	PAL-2	Hesperetin	C01709	0.907*
3	TRINITY_DN2373_c0_g4	PAL-2	Dihydrokaempferol	C00974	0.907*
4	TRINITY_DN2373_c0_g4	PAL-2	Cyanidin 5-O-beta-D-glucoside 3-O-beta-D-sambubioside	C20493	0.904*
5	TRINITY_DN2373_c0_g4	PAL-2	Cyanidin 3-O-(2-O-beta-D-glucuronosyl)-beta-D-glucoside	C19762	-0.933*
6	TRINITY_DN2373_c0_g4	PAL-2	Pelargonidin 3-O-glucoside	C12137	-0.947*
7	TRINITY_DN2373_c0_g2	PAL-1	Liquiritigenin	C09762	-0.992**
8	TRINITY_DN2373_c0_g2	PAL-1	Hesperetin	C01709	0.919*
9	TRINITY_DN2373_c0_g2	PAL-1	Epicatechin	C09727	-0.915*
10	TRINITY_DN2373_c0_g2	PAL-1	Myricetin	C10107	-0.943*
11	TRINITY_DN2373_c0_g2	PAL-1	Cyanidin 5-O-beta-D-glucoside 3-O-beta-D-sambubioside	C20493	0.912*
12	TRINITY_DN2373_c0_g2	PAL-1	Malvidin-3-(p-coumaroyl)-rutinoside-5-glucoside	C16296	-0.954*
13	TRINITY_DN2373_c0_g2	PAL-1	Cyanidin 3-O-(2-O-beta-D-glucuronosyl)-beta-D-glucoside	C19762	-0.960**
14	TRINITY_DN2373_c0_g2	PAL-1	Pelargonidin 3-O-glucoside	C12137	-0.945*
15	TRINITY_DN43293_c0_g1	IF7MAT	Neohesperidin	C09806	0.897*
16	TRINITY_DN43293_c0_g1	IF7MAT	Naringin	C09789	-0.927*
17	TRINITY_DN43293_c0_g1	IF7MAT	Malvidin-3-(p-coumaroyl)-rutinoside-5-glucoside	C16296	-0.901*
18	TRINITY_DN43293_c0_g1	IF7MAT	Peonidin-3-(p-coumaroyl)-rutinoside-5-glucoside	C16293	0.984**
19	TRINITY_DN43293_c0_g1	IF7MAT	Pelargonidin 3-O-(6-caffeoyl-beta-D-glucoside)	C16297	0.932*
20	TRINITY_DN5804_c0_g1	HCT-2	Prunin	C09099	0.970**
21	TRINITY_DN5804_c0_g1	HCT-2	Pelargonidin 5-O-beta-D-glucoside 3-O-beta-D-sambubioside	C20492	-0.952*
22	TRINITY_DN43793_c0_g1	HCT-1	Neohesperidin	C09806	-0.978**
23	TRINITY_DN43793_c0_g1	HCT-1	Cyanidin 3-O-beta-D-sambubioside	C20490	-0.908*
24	TRINITY_DN43793_c0_g1	HCT-1	Malvidin-3-(p-coumaroyl)-rutinoside-5-glucoside	C16296	0.977**
25	TRINITY_DN43793_c0_g1	HCT-1	Pelargonidin 3-O-(6-caffeoyl-beta-D-glucoside)	C16297	-0.959*
26	TRINITY_DN7300_c0_g1	FLS	Naringenin 7-O-beta-D-glucoside	C09099	-0.913*
27	TRINITY_DN759_c1_g1	CYP81E	Neohesperidin	C09806	0.911*
28	TRINITY_DN759_c1_g1	CYP81E	(-)-Epicatechin	C09727	-0.942*
29	TRINITY_DN759_c1_g1	CYP81E	2',3,4,4',6'-Peptahydroxychalcone 4'-O-glucoside	C16408	-0.895*
30	TRINITY_DN759_c1_g1	CYP81E	Xanthohumol	C16417	-0.907*
31	TRINITY_DN759_c1_g1	CYP81E	Myricetin	C10107	-0.978**
32	TRINITY_DN759_c1_g1	CYP81E	Malvidin-3-(p-coumaroyl)-rutinoside-5-glucoside	C16296	-0.978**

33	TRINITY_DN759_c1_g1	CYP81E	Pelargonidin 3-O-(6-caffeoyl-beta-D-glucoside)	C16297	0.882*
34	TRINITY_DN18327_c0_g1	CYP81E	Prunin	C09099	-0.916*
35	TRINITY_DN18327_c0_g1	CYP81E	Dihydrokaempferol	C00974	0.993**
36	TRINITY_DN18327_c0_g1	CYP81E	Cyanidin 5-O-beta-D-glucoside 3-O-beta-D-sambubioside	C20493	0.962**
37	TRINITY_DN16239_c0_g1	CYP75A-1	Malvidin-3-(p-coumaroyl)-rutinoside-5-glucoside	C16296	0.939*
38	TRINITY_DN16239_c0_g1	CYP75A-1	Peonidin 3-O-glucoside	C12141	-0.969**
39	TRINITY_DN37189_c0_g1	CYP73A-2	Pinostrobin	C16419	-0.966**
40	TRINITY_DN37189_c0_g1	CYP73A-2	Phlorizin	C01604	0.998**
41	TRINITY_DN37189_c0_g1	CYP73A-2	Pelargonidin 3-O-rutinoside 5-O-beta-D-glucoside	C12645	-0.959**
42	TRINITY_DN37189_c0_g2	CYP73A	Prunin	C09099	-0.924*
43	TRINITY_DN37189_c0_g2	CYP73A	Liquiritigenin	C09762	-0.903*
44	TRINITY_DN37189_c0_g2	CYP73A	Naringenin 7-O-beta-D-glucoside	C09099	-0.970**
45	TRINITY_DN37189_c0_g2	CYP73A	Dihydrokaempferol	C00974	0.900*
46	TRINITY_DN37189_c0_g2	CYP73A	Pelargonidin 5-O-beta-D-glucoside 3-O-beta-D-sambubioside	C20492	0.901*
47	TRINITY_DN37189_c0_g2	CYP73A	Cyanidin 3-O-(2-O-beta-D-glucuronosyl)-beta-D-glucoside	C19762	-0.913*
48	TRINITY_DN37189_c0_g2	CYP73A	Pelargonidin 3-O-glucoside	C12137	-0.961**
49	TRINITY_DN341_c2_g2	CYP71D9	Prunin	C09099	0.95*
50	TRINITY_DN341_c2_g2	CYP71D9	Naringin	C09789	0.879*
51	TRINITY_DN341_c2_g2	CYP71D9	Dihydrokaempferol	C00974	-0.968**
52	TRINITY_DN341_c2_g2	CYP71D9	Cyanidin 5-O-beta-D-glucoside 3-O-beta-D-sambubioside	C20493	-0.959**
53	TRINITY_DN9119_c0_g1	CCOAOMT-4	Liquiritigenin	C09762	-0.909*
54	TRINITY_DN9119_c0_g1	CCOAOMT-4	Hesperetin	C01709	0.900*
55	TRINITY_DN1098_c1_g2	CCOAOMT-3	Neohesperidin	C09806	-0.935*
56	TRINITY_DN1098_c1_g2	CCOAOMT-3	Phlorizin chalcone	C16406	0.919*
57	TRINITY_DN1098_c1_g2	CCOAOMT-3	Phlorizin	C01604	0.930*
58	TRINITY_DN1098_c1_g2	CCOAOMT-3	Cyanidin 3-O-beta-D-sambubioside	C20490	-0.963**

59	TRINITY_DN1098_c1_g2	CCOAOMT-3	Cyanidin 3-O-rutinoside 5-O-beta-D-glucoside	C12646	0.926*
60	TRINITY_DN8199_c3_g1	CCOAOMT-2	Phlorizin	C01604	0.948*
61	TRINITY_DN8199_c3_g1	CCOAOMT-2	Pelargonidin	C05904	0.918*
62	TRINITY_DN8199_c3_g1	CCOAOMT-2	Cyanidin 3-glucoside 5-caffeoylglucoside	C16372	0.967**
63	TRINITY_DN8199_c3_g1	CCOAOMT-2	Pelargonidin 3-O-rutinoside 5-O-beta-D-glucoside	C12645	-0.947*
64	TRINITY_DN8199_c3_g1	CCOAOMT-2	Peonidin-3-(p-coumaroyl)-rutinoside-5-glucoside	C16293	0.951*
65	TRINITY_DN8199_c3_g1	CCOAOMT-2	Petunidin 3-O-glucoside	C12139	0.989**
66	TRINITY_DN19637_c0_g1	CCOAOMT-1	2',3,4,4',6'-Peptahydroxychalcone 4'-O-glucoside	C16408	0.952*
67	TRINITY_DN19637_c0_g1	CCOAOMT-1	Phlorizin	C01604	0.919*
68	TRINITY_DN19637_c0_g1	CCOAOMT-1	Cyanidin 3-glucoside 5-caffeoylglucoside	C16372	0.949*
69	TRINITY_DN19637_c0_g1	CCOAOMT-1	Cyanidin 3-O-beta-D-sambubioside	C20490	-0.929*
70	TRINITY_DN19637_c0_g1	CCOAOMT-1	Pelargonidin 3-O-rutinoside 5-O-beta-D-glucoside	C12645	-0.902*
71	TRINITY_DN19637_c0_g1	CCOAOMT-1	Petunidin 3-O-glucoside	C12139	0.986**
72	TRINITY_DN3551_c0_g1	C12RT1	Neohesperidin	C09806	0.912*
73	TRINITY_DN3551_c0_g1	C12RT1	Cyanidin 5-O-beta-D-glucoside 3-O-beta-D-sambubioside	C20493	0.894*
74	TRINITY_DN3551_c0_g1	C12RT1	Malvidin-3-(p-coumaroyl)-rutinoside-5-glucoside	C16296	-0.977**
75	TRINITY_DN3551_c0_g1	C12RT1	Peonidin 3-O-glucoside	C12141	0.959**
76	TRINITY_DN3551_c0_g1	C12RT1	Pelargonidin 3-O-(6-caffeoyl-beta-D-glucoside)	C16297	0.946*
77	TRINITY_DN6675_c0_g1	4CL	Hesperetin 7-O-glucoside	C16422	0.951*

Table S8 Transcription factor families with current 10 gene numbers

Transcription factor families	gene numbers
C2H2	34
bZIP	27
bHLH	27
MYB_related	27
GRAS	25
ERF	24
NAC	20

FAR1	20
MYB	18

Table S9 Correlation of "Transcription factor - Flavonoid differentially expressed genes" in the leaves of *Ludisia discolor*

No	Regulated gene	IF	Enzyme gene		Correlation
1	TRINITY_DN10318_c0_g1	bZIP53	TRINITY_DN2373_c0_g4	PAL-2	-0.907*
2	TRINITY_DN10318_c0_g1	bZIP53	TRINITY_DN37189_c0_g2	CYP73A	-0.913*
3	TRINITY_DN10318_c0_g1	bZIP53	TRINITY_DN341_c2_g2	CYP71D9	0.979**
4	TRINITY_DN10318_c0_g1	bZIP53	TRINITY_DN18327_c0_g1	CYP81E-1	-0.979**
5	TRINITY_DN11409_c0_g1	bZIP2	TRINITY_DN1098_c1_g2	CCOAOMT-3	-0.915*
6	TRINITY_DN11409_c0_g1	bZIP2	TRINITY_DN43793_c0_g1	HCT-1	-0.928*
7	TRINITY_DN11409_c0_g1	bZIP2	TRINITY_DN759_c1_g1	CYP81E-2	0.942*
8	TRINITY_DN1499_c0_g1	bHLH4	TRINITY_DN2373_c0_g2	PAL-1	-0.902*
9	TRINITY_DN1499_c0_g1	bHLH4	TRINITY_DN16239_c0_g1	CYP75A	0.974**
10	TRINITY_DN1499_c0_g1	bHLH4	TRINITY_DN3551_c0_g1	C12RT1	-0.997**
11	TRINITY_DN1499_c0_g1	bHLH4	TRINITY_DN43793_c0_g1	HCT-1	0.948*
12	TRINITY_DN1499_c0_g1	bHLH4	TRINITY_DN43293_c0_g1	IF7MAT	-0.981**
13	TRINITY_DN1743_c1_g1	bZIP44-1	TRINITY_DN2373_c0_g2	PAL-1	-0.939*
14	TRINITY_DN1743_c1_g1	bZIP44-1	TRINITY_DN5804_c0_g1	HCT-2	0.923*
15	TRINITY_DN1743_c1_g2	bZIP44-2	TRINITY_DN1098_c1_g2	CCOAOMT-3	-0.982**
16	TRINITY_DN1743_c1_g2	bZIP44-2	TRINITY_DN43793_c0_g1	HCT-1	-0.955*
17	TRINITY_DN1743_c2_g1	bZIP44-3	TRINITY_DN2373_c0_g4	PAL-2	0.939*
18	TRINITY_DN1743_c2_g1	bZIP44-3	TRINITY_DN2373_c0_g2	PAL-1	0.986**
19	TRINITY_DN1743_c2_g1	bZIP44-3	TRINITY_DN3551_c0_g1	C12RT1	0.945*
20	TRINITY_DN1743_c2_g1	bZIP44-3	TRINITY_DN9119_c0_g1	CCOAOMT-4	0.913*
21	TRINITY_DN2543_c0_g1	MYBCD5	TRINITY_DN341_c2_g2	CYP71D9	0.981**
22	TRINITY_DN2543_c0_g1	MYBCD5	TRINITY_DN18327_c0_g1	CYP81E-1	-0.983**
23	TRINITY_DN3701_c0_g1	bHLH16	TRINITY_DN4773_c2_g1	4CL-1	0.95*
24	TRINITY_DN3701_c0_g1	bHLH16	TRINITY_DN7300_c0_g1	FLS	0.963**
25	TRINITY_DN4112_c0_g1	bZIP36-2	TRINITY_DN16239_c0_g1	CYP75A	0.95*
26	TRINITY_DN4112_c0_g1	bZIP36-2	TRINITY_DN3551_c0_g1	C12RT1	-0.966**
27	TRINITY_DN4112_c0_g1	bZIP36-2	TRINITY_DN43793_c0_g1	HCT-1	0.988**
28	TRINITY_DN4112_c0_g1	bZIP36-2	TRINITY_DN759_c1_g1	CYP81E-2	-0.935*
29	TRINITY_DN4112_c0_g1	bZIP36-2	TRINITY_DN43293_c0_g1	IF7MAT	-0.939*
30	TRINITY_DN5177_c0_g1	MYB88	TRINITY_DN7300_c0_g1	FLS	-0.981**
31	TRINITY_DN6460_c0_g1	MYB93	TRINITY_DN2373_c0_g4	PAL-2	0.907*
32	TRINITY_DN6460_c0_g1	MYB93	TRINITY_DN2373_c0_g2	PAL-1	0.979**
33	TRINITY_DN6460_c0_g1	MYB93	TRINITY_DN37189_c0_g2	CYP73A	0.924*
34	TRINITY_DN7148_c0_g1	bHLH13	TRINITY_DN6675_c0_g1	4CL-2	0.978**
35	TRINITY_DN9_c0_g2	bZIP36-4	TRINITY_DN2373_c0_g4	PAL-2	-0.942*
36	TRINITY_DN9_c0_g2	bZIP36-4	TRINITY_DN37189_c0_g2	CYP73A	-0.959**

37	TRINITY_DN9_c0_g2	bZIP36-4	TRINITY_DN9119_c0_g1	CCOAOMT-4	-0.906*

Note: * p＜0.05，significant correlation; ** p＜0.01，extremely significant correlation.

Table S10 Correlation of "Endogenous hormone - Transcription factor"in the leaves of *Ludisia discolor*

NO	Endogenous hormone	Regulator gene	Name	Correlation
1	IAA	TRINITY_DN1743_c1_g2	bZIP44-2	-0.907*
2	GA24	TRINITY_DN2543_c0_g1	MYBCD5	-0.925*
3	GA24	TRINITY_DN1499_c0_g1	BHLH4	-0.959**
4	GA24	TRINITY_DN6460_c0_g1	MYB93	0.951*
5	GA24	TRINITY_DN10318_c0_g1	BZIP53	-0.933*
6	GA24	TRINITY_DN1743_c1_g1	bZIP44-1	-0.924*
7	GA24	TRINITY_DN1743_c2_g1	bZIP44-3	0.995**
8	GA4	TRINITY_DN5177_c0_g1	MYB88	-0.958*
9	GA4	TRINITY_DN3701_c0_g1	BHLH16	0.922*
10	GA36	TRINITY_DN2543_c0_g1	MYBCD5	-0.973**
11	GA36	TRINITY_DN6460_c0_g1	MYB93	0.94*
12	GA36	TRINITY_DN10318_c0_g1	BZIP53	-0.991**
13	GA36	TRINITY_DN1743_c2_g1	bZIP44-3	0.933*
14	GA36	TRINITY_DN9_c0_g2	BZIP36-4	-0.911*
15	GA5	TRINITY_DN1743_c1_g2	bZIP44-2	-0.955*
16	GA7	TRINITY_DN1276_c1_g1	BZIP36-1	-0.992**
17	GA8	TRINITY_DN5177_c0_g1	MYB88	0.982**
18	GA20	TRINITY_DN1743_c1_g2	bZIP44-2	-0.907*
19	GA20	TRINITY_DN1276_c1_g1	BZIP36-1	-0.984**
20	ACC	TRINITY_DN7148_c0_g1	BHLH13	0.938*
21	DZR	TRINITY_DN8596_c0_g1	BHLH78	0.949*
22	DHZ	TRINITY_DN1743_c1_g1	bZIP44-1	0.927*
23	DHZ	TRINITY_DN1276_c1_g1	BZIP36-1	-0.94*
24	tZT	TRINITY_DN1743_c1_g2	bZIP44-2	-0.993**
25	tZT	TRINITY_DN4112_c0_g1	BZIP36-2	0.95*
26	tZT	TRINITY_DN11409_c0_g1	bZIP2	-0.947*
27	ZT	TRINITY_DN1743_c1_g1	bZIP44-1	0.905*
28	ZT	TRINITY_DN1743_c1_g2	bZIP44-2	-0.911*
29	ZT	TRINITY_DN11409_c0_g1	bZIP2	-0.962**
30	cZR	TRINITY_DN2543_c0_g1	MYBCD5	-0.993**
31	cZR	TRINITY_DN6460_c0_g1	MYB93	0.934*
32	cZR	TRINITY_DN10318_c0_g1	BZIP53	-0.997**
33	cZR	TRINITY_DN1743_c1_g1	bZIP44-1	-0.901*
34	cZR	TRINITY_DN1743_c2_g1	bZIP44-3	0.953*
35	ABA	TRINITY_DN1499_c0_g1	BHLH4	0.95*
36	ABA	TRINITY_DN1743_c2_g1	bZIP44-3	-0.92*
37	ABA	TRINITY_DN4112_c0_g1	BZIP36-2	0.917*
38	BR	TRINITY_DN1743_c1_g2	bZIP44-2	-0.984**

39	BR	TRINITY_DN4112_c0_g1	BZIP36-2	0.934*
40	BR	TRINITY_DN11409_c0_g1	bZIP2	-0.964**

Table S11 Correlation of "Endogenous hormone - Transcription factor"in the leaves of *Ludisia discolor*

NO	Endogenous hormone	Regulator gene	Name	Correlation
1	IAA	TRINITY_DN1743_c1_g2	bZIP44-2	-0.907*
2	GA24	TRINITY_DN2543_c0_g1	MYBCD5	-0.925*
3	GA24	TRINITY_DN1499_c0_g1	bHLH4	-0.959**
4	GA24	TRINITY_DN6460_c0_g1	MYB93	0.951*
5	GA24	TRINITY_DN10318_c0_g1	bZIP53	-0.933*
6	GA24	TRINITY_DN1743_c1_g1	bZIP44-1	-0.924*
7	GA24	TRINITY_DN1743_c2_g1	bZIP44-3	0.995**
8	GA4	TRINITY_DN5177_c0_g1	MYB88	-0.958*
9	GA4	TRINITY_DN3701_c0_g1	bHLH16	0.922*
10	GA36	TRINITY_DN2543_c0_g1	MYBCD5	-0.973**
11	GA36	TRINITY_DN6460_c0_g1	MYB93	0.940*
12	GA36	TRINITY_DN10318_c0_g1	bZIP53	-0.991**
13	GA36	TRINITY_DN1743_c2_g1	bZIP44-3	0.933*
14	GA36	TRINITY_DN9_c0_g2	bZIP36-4	-0.911*
15	GA5	TRINITY_DN1743_c1_g2	bZIP44-2	-0.955*
16	GA7	TRINITY_DN1276_c1_g1	bZIP36-1	-0.992**
17	GA8	TRINITY_DN5177_c0_g1	MYB88	0.982**
18	GA20	TRINITY_DN1743_c1_g2	bZIP44-2	-0.907*
19	GA20	TRINITY_DN1276_c1_g1	bZIP36-1	-0.984**
20	ACC	TRINITY_DN7148_c0_g1	bHLH13	0.938*
21	DZR	TRINITY_DN8596_c0_g1	bHLH13	0.949*
22	DHZ	TRINITY_DN1743_c1_g1	bZIP44-1	0.927*
23	DHZ	TRINITY_DN1276_c1_g1	bZIP36-1	-0.940*
24	tZT	TRINITY_DN1743_c1_g2	bZIP44-2	-0.993**
25	tZT	TRINITY_DN4112_c0_g1	bZIP36-2	0.950*
26	tZT	TRINITY_DN11409_c0_g1	bZIP2	-0.947*
27	ZT	TRINITY_DN1743_c1_g1	bZIP44-1	0.905*
28	ZT	TRINITY_DN1743_c1_g2	bZIP44-2	-0.911*
29	ZT	TRINITY_DN11409_c0_g1	bZIP2	-0.962**
30	cZR	TRINITY_DN2543_c0_g1	MYBCD5	-0.993**
31	cZR	TRINITY_DN6460_c0_g1	MYB93	0.934*
32	cZR	TRINITY_DN10318_c0_g1	bZIP53	-0.997**
33	cZR	TRINITY_DN1743_c1_g1	bZIP44-1	-0.901*
34	cZR	TRINITY_DN1743_c2_g1	bZIP44-3	0.953*
35	ABA	TRINITY_DN1499_c0_g1	bHLH4	0.950*
36	ABA	TRINITY_DN1743_c2_g1	bZIP44-3	-0.920*
37	ABA	TRINITY_DN4112_c0_g1	bZIP36-2	0.917*
38	BR	TRINITY_DN1743_c1_g2	bZIP44-2	-0.984**
39	BR	TRINITY_DN4112_c0_g1	bZIP36-2	0.934*

Note: * $p < 0.05$, significant correlation; ** $p < 0.01$, extremely significant correlation.

Table S12 Conditions of flavonoids-related transcription factors in the leaves of *Ludisia discolor*

IF	Hormone response elements	Core sequence	Endogenous hormone	Number
MYBCD5	P-box	CCTTTTG	GA	1
MYB88	TGACG-motif	TGACG	Me_JA	2
MYB88	CGTCA-motif	CGTCA	Me_JA	2
MYB88	TGA-element	AACGAC	AUX	2
MYB88	GARE-motif	TCTGTTG	GA	2
MYB88	P-box	CCTTTTG	GA	1
MYB93	CGTCA-motif	CGTCA	Me_JA	4
MYB93	TGACG-motif	TGACG	Me_JA	4
MYB93	TGA-element	AACGAC	AUX	2
bHLH4	ABRE	ACGTG	ABA	2
bHLH4	TGA-element	AACGAC	AUX	1
bHLH4	TATC-box	TATCCCA	GA	2
bHLH6	TGACG-motif	TGACG	Me_JA	2
bHLH6	CGTCA-motif	CGTCA	Me_JA	2
bHLH6	P-box	CCTTTTG	GA	2
bHLH13	GARE-motif	TCTGTTG	GA	1
bZIP2	TGACG-motif	TGACG	Me_JA	3
bZIP2	CGTCA-motif	CGTCA	Me_JA	3
bZIP2	ABRE	AACCCGG	ABA	3
bZIP2	TGA-element	AACGAC	AUX	1
bZIP2	TATC-box	TATCCCA	GA	2
bZIP36	TGACG-motif	TGACG	Me_JA	1
bZIP36	CGTCA-motif	CGTCA	Me_JA	1
bZIP36	AuxRR-core	GGTCCAT	AUX	1
bZIP44	CGTCA-motif	CGTCA	Me_JA	2
