# Supplementary figures and images for: Multi-Omics Reveals Light-Quality-Dependent Phytohormone and Transcription Factor Networks Regulating Flavonoid Biosynthesis in Ludisia discolor
Source: Genes (Basel). 2026 Apr 13;17(4):445. doi: 10.3390/genes17040445 (PMC13115993; doi:10.3390/genes17040445)

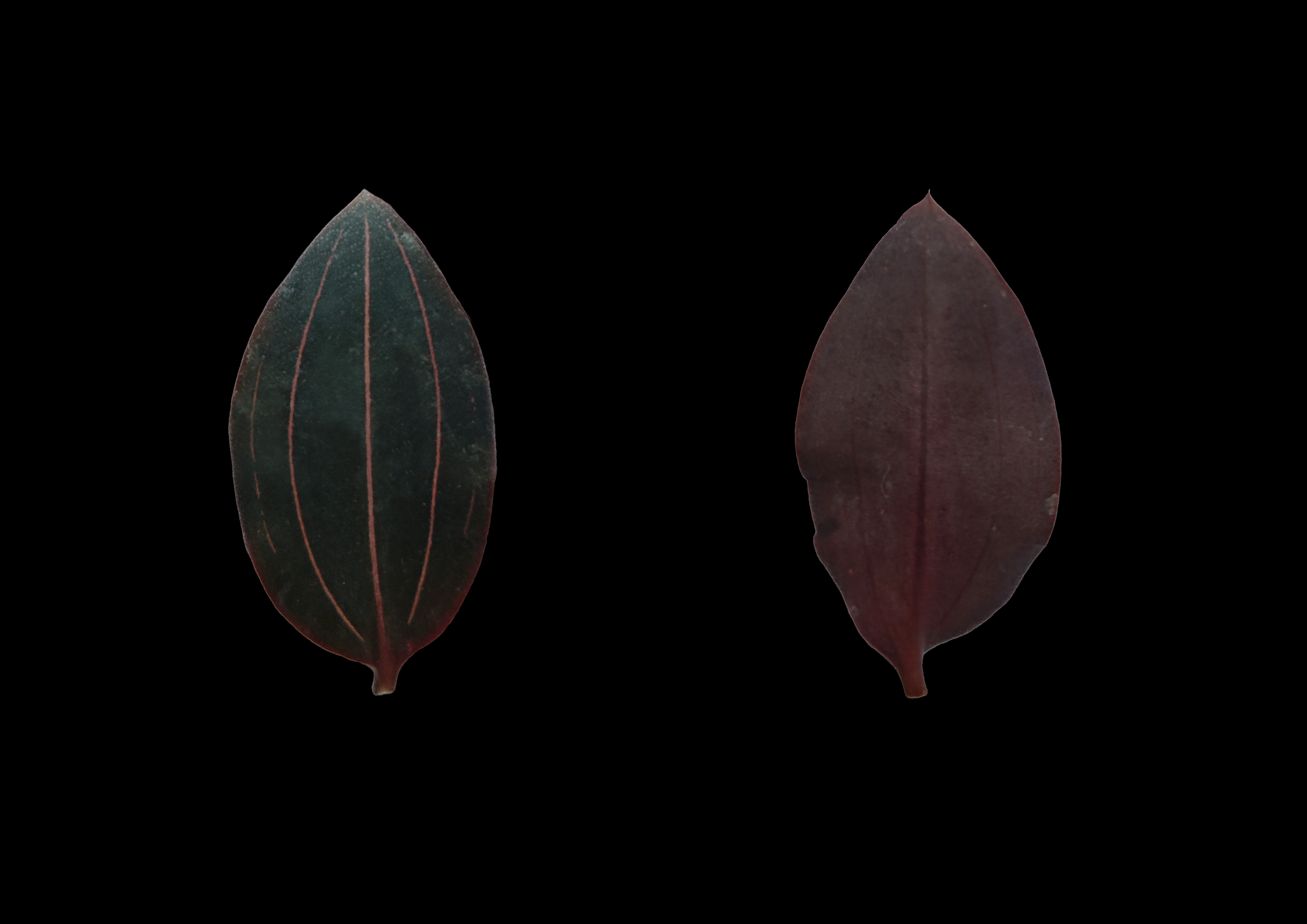

Supplement: Supplementary file 1 [file genes-17-00445-s001.zip › Figure S1 Leaves of ’Min Hot Round Shuai'.jpg]
